# Examining the Alterations in Metabolite Constituents and Antioxidant Properties in Mountain-Cultivated Ginseng (*Panax ginseng* C.A. Meyer) Organs during a Two-Month Maturation Period

**DOI:** 10.3390/antiox13050612

**Published:** 2024-05-17

**Authors:** Hee Yul Lee, Du Yong Cho, Du Hyun Kim, Jong-Hwan Park, Jong Bin Jeong, Se Hyeon Jeon, Ji Ho Lee, Eun Jeong Ko, Kye Man Cho, Jin Hwan Lee

**Affiliations:** 1 Department of Green Bio Science and Agri-Food Bio Convergence Institute, Gyeongsang National University, Jinju 52725, Republic of Korea; 2 Department of Life Resource Industry, Dong-A University, 37, Nakdong-Daero 550 Beon-gil, Saha-gu, Busan 49315, Republic of Korea

**Keywords:** mountain-cultivated ginseng, organ, maturation time, metabolite, antioxidant, leaves

## Abstract

The current research was the first to prove the existence of fluctuations in the metabolite constituents and antioxidant properties in different organs (leaves, stems, and roots) of the mountain-cultivated ginseng (MCG) plant during a two-month maturation period. Four metabolites, including fatty acids, amino acids, ginsenosides, and phenolic phytochemicals, exhibited considerable differences in organs and maturation times with the following order: leaves > stems > roots. The predominant metabolite contents were found in leaves, with fatty acid (1057.9 mg/100 g) on 31 May, amino acid (1989.2 mg/100 g) on 13 July, ginsenosides (88.7 mg/g) on 31 May, and phenolic phytochemical (638.3 μg/g) on 31 May. Interestingly, ginsenoside content in leaves were highest, with 84.8 → 88.7 → 82.2 → 78.3 mg/g. Specifically, ginsenosides Re, Rd, and F2 showed abundant content ranging from 19.1 to 16.9 mg/g, 8.5 to 14.8 mg/g, and 9.5 to 13.1 mg/g, respectively. Phenolic phytochemicals exhibited remarkable differences in organs compared to maturation periods, with the highest total phenolic content and total flavonoid content recorded at 9.48 GAE and 1.30 RE mg/g in leaves on 31 May. The antioxidant capacities on radical, FRAP, and DNA protection differed significantly, with leaves on 31 May exhibiting the highest values: 88.4% (DPPH), 89.5% (ABTS), 0.84 OD593 nm (FRAP) at 500 μg/mL, and 100% DNA protection at 50 μg/mL. Furthermore, principal cluster analysis revealed metabolite variability as follows: ginsenoside (83.3%) > amino acid (71.8%) > phenolic phytochemical (61.1%) > fatty acid (58.8%). A clustering heatmap highlighted significant changes in metabolite components under the maturation times for each organ. Our findings suggest that MCG leaves on 31 May may be a potential source for developing nutraceuticals, offering highly beneficial components and strong antioxidants.

## 1. Introduction

Ginseng (*Panax ginseng* C.A. Meyer) is a perennial plant belonging to the Panax genus of the Araliaceae family, and for several decades it has been widely used as herbal medicine in East Asia, particularly in Korea, China, and Japan [1,2,3,4,5]. This species is commonly classified into three types based on the cultivated method and growth environment, as follows: cultivated ginseng (CG), mountain-cultivated ginseng (MCG), and mountain-wild ginseng (MWG) [6,7,8]. Previously, many researchers have reported that ginseng possesses diverse nutritional constituents, including organic acids, vitamins, amino acids, polysaccharides, phenolic compounds, and ginsenosides [3,6,8,9,10,11]. Among them, ginsenosides known as triterpene saponins have a tetracyclic structure, and are divided into protopanaxadiol (PPD) (ginsenoside Rb1, Rc, Rb2, Rb3, Rd, Rd2, Rg3, Rh2, F2, etc.), protopanaxatriol (PPT) (ginsenoside Rg1, Re, Rf, Rg2, Rh1, F1, F3, F5, etc.), and oleanane (Ro) forms [1,2,3,6,12]. This metabolite has beneficial properties, namely, antioxidant, anti-inflammatory, antidiabetic, anticancer, and immunity enhancements in the field of human health [1,4,6,12,13,14]. Moreover, this constituent contains abundant value derivatives such as ginsenoside Rb1, Rh2, Rc, Rd, Re, Rf, and Rg1 [1,5,15], and minor ginsenosides (Rg2, Rg3, Rh1, F1, F2, and compound K) have revealed higher biological effects than those in the main ginsenosides [4,12]. In particular, the phenolic metabolites in ginseng, such as ferulic, caffeic, syringic, vanillic, and gentisic acids, demonstrated anticancer and antioxidant abilities [6,11,13,16]. Other nutritional metabolites, such as amino acids and fatty acids, also play essential roles in health-promoting functional characteristics regarding lipid deposition, protein synthesis, pharmacological capacities of antiatherosclerosis, and the prevention of chronic diseases [11,17,18]. Interestingly, ginsenoside compositions are distributed in organs (roots, stems, flowers, leaves, and rhizomes) of Panax botanicals, and their ratios in leaves and stems (3–6%) are higher than those in other organs, including roots (1–3%) [19,20]. Most specifically, recent studies have revealed that ginseng leaves exhibited approximately four-fold higher content compared to roots and stems, and their total ginsenoside distributions were in the following order: leaves ˃ stems ˃ roots [21,22]. Accordingly, the appropriate growth times and organs of ginseng are very important for obtaining high metabolite content and strong biological properties for medicinal and functional food applications concerning human dietary supplements and nutritional values. Although metabolite concentrations and distributions exhibited considerable differences in ginseng sources according to multiple factors, including processing skills [2,3,9,10,15,23], no published information on the fluctuations of various metabolites at growth periods through environmental parameters is available. Moreover, few studies have examined the variations in antioxidant functions in radical and DNA protection from ginseng organs at different maturation times.

Recently, we documented the changes in ginsenosides and the biological effects of ginseng sprouts through aging and fermentation techniques [15]. In our ongoing exploration of function properties, MCG organs observed significant differences in metabolite constituents and antioxidant capacities. Therefore, our work was conducted to investigate the various metabolite content, along with the antioxidant properties in MCG organs across distinct maturation times.

The primary aim of this study was to search for effective organs and appropriate maturation times with not only high nutritional metabolites, but also strong antioxidant properties for the development of functional sources from MCG. Herein, for the first time, changes in the content of four metabolite constituents (fatty acids, amino acids, ginsenosides, and phenolic phytochemicals) from MCG organs (leaves, stems, and roots) were examined and compared through different maturation periods. By comprehensively comparing and analyzing the results, considering the content of ginsenosides as an essential parameter, this study aims to find the appropriate maturation times. In addition, this study documented the antioxidant properties (radical, FRAP, and DNA protection) and substances (total phenolic contents, TPC, and total flavonoid contents, TFC) and analyzed their correlations with metabolites through the maturation times in the organs of this plant.

## 2. Materials and Methods

### 2.1. Plant Sources and Chemicals

MCG was sown on 15 April 2017 in the experimental field at Simmani Wild Ginseng Farm Association Co. (Seoha-myeon, Hamyang-gun, Gyeongsangnam province, Republic of Korea). Three organs of the MCG plant (5-year-old), namely, leaves, stems, and roots, were collected at four different maturation times as follows: 1st harvest (17 May 2022), 2nd harvest (31 May 2022), 3rd harvest (21 June 2022), and 4th harvest (13 July 2022) (Figure S1). The collected MCG sources were determined according to the maturity rates (optimal periods: between May and July) of 5- or 6-year-old MCG plants for the development of processed foods using ginseng in Korea. After sampling, the MCG materials were carefully washed with deionized water to remove the dust residual, and then air-dried at 25 °C for three days. The dried MCG sample was chopped according to each organ and stored at −10 °C before analysis. The general appearances of MCG through maturation times are displayed in the Supporting Materials.

For the antioxidant assays, 2,2-diphenyl-1-pycrylhydrazyl (DPPH), 2,2′-azino-bis(3-ethylbenzthiazoline-6-sulphonic acid) (ABTS), 4,6-tripyridyl-s-triazine (TPTZ), butylated hydroxytoluene (BHT), 6-hydroxy-2,5,7,8-tetramethylchroman-2-carboxylic acid (Trolox), and ascorbic acid were acquired from Sigma-Aldrich Chemical Co. (St. Louis, MO, USA). Folin–Ciocalteu phenol reagent, gallic acid, rutin, potassium persulphate, and diethylene glycol were also obtained from Sigma-Aldrich Chemical. The fatty acid, amino acid, ginsenoside (ginsenoside Rb1, Rb2, Rb3, Rg1, Rg2, Rg3, Rd, Rd2, F1, F2, F3, F5, Rh1, Rh2, Rc, Re, Rf, and Ro, compound K, protopanaxdiol, and protopanaxtriol), and phenolic phytochemical standards were obtained from Sigma-Aldrich Chemical Co. and KOC Biotech Co., Ltd. (Daejeon, Republic of Korea). Analytical grade water, acetonitrile, and acetic acid were purchased for high-performance liquid chromatography (HPLC) analysis from J.T. Baker (Phillipsburg, NJ, USA). Other chemicals and solvents were processed using the analytical grade without purification.

### 2.2. Instruments

The antioxidant abilities, TPC and TFC, were measured from UV-Vis spectra using Agilent BioTek spectrophotometry (EPOCH 2, Winooski, VT, USA). The fatty acid components were evaluated using gas chromatography (GC) (Agilent Technologies 7980 system, Wilmington, DE, USA) coupled to a flame ion detector with an SP-2560 capillary column (100 m × 0.25 mm i.d., 0.25 μm film thickness, St. Louis, MO, USA). The amino acid contents were demonstrated using an amino acid analyzer (L-8900, Hitachi High-Technologies, Tokyo, Japan). Other metabolites were conducted using an Agilent 1260 system (Waldbronn, Germany) consisting of an Agilent 1260 diode-array detector, autosampler, and quaternary pump with a TSK-ODS100Z column (4.6 × 250 mm, 5 μm, Tosoh Corp., Tokyo, Japan) for ginsenosides and X-Bridge C18-RP column (4.6 × 250 mm, 5 μm, Waters Corp., Milford, MA, USA) for phenolic phytochemicals.

### 2.3. Weight of Organs (Such as Leaves, Stems, and Roots)

First, 100 plants were randomly selected from each of the washed samples using harvest dates. The selected samples were divided into each part (leaves, stems, and roots), and the weight of fresh was measured. Then, fresh samples were dried for 48 h at 50 °C, and dried samples were measured. The values, fresh and dried organ weight, were expressed as average organ ratio per MCG.

### 2.4. Preparation of Extract Concentrates

One gram of the sample powder was combined with 20 mL of 50% ethanol, and this mixture was subjected to extraction at 40 ± 2 °C for 2 h. To separate the supernatant, the resulting extract was then passed through a 0.45 µm membrane filter. This filtration step was performed twice. Additionally, for the experiments, we prepared three extracts from each sample using the same method. Created concentrated extracts were used for the analyses of TPC, TFC, antioxidant activities, and DNA protection capacity analysis.

### 2.5. TPC and TFC Analyses

The TPC and TFC were measured using spectrophotometry [15,24]. TPC was evaluated colorimetrically using the Folin–Ciocalteu reagent. In short, the appropriately diluted MCG extract (50% ethanol, 500 μL) or positive control (gallic acid; 500 μL) was mixed with 1 N Folin–Ciocalteu reagent (250 μL), and the sodium carbonate solution (20%, 500 μL) was then added to the Folin–Ciocalteu mixture. After incubation at 35 °C for 10 min, absorbance was evaluated at 750 nm using a spectrophotometer and compared with a calibration curve (0.01–1 mg/mL). All contents were confirmed as gallic acid equivalents (GAE) per milligram of dried MCG sample (GAE mg/g). TFC was also documented using the colorimetric method. The MCG sample or rutin (positive control; 250 μL) was added to 2 mL of 50% ethanol, followed by 90% diethylene glycol (1 mL) and 4 M NaOH (1 mL). After heating for 10 min at 35 °C, the crude solution was measured at 420 nm and compared with a calibration curve (rutin, 0.01–1 mg/mL). The results were expressed as milligram equivalents of rutin per 1 g of the dried MCG sample (RE mg/g).

### 2.6. Antioxidant Activities Based on Radical and FRAP Assays

Radical scavenging and FRAP methods were selected for the analyses of antioxidant effects, and their experimental techniques were performed using the earlier-reported procedures with some modifications [3,6,23]. Radical scavenging assays and FRAP assays were performed in triplicates. To measure the DPPH scavenging abilities, a solution of 1 mM DPPH was adjusted to 0.70 at 517 nm, and 50% ethanol extract (100 μL, MCG sample) or BHT (positive control, 100 μL) with diverse concentrations was added with 1 mM DPPH solution (3.9 mL). The crude solution was maintained for 30 min at 25 °C in darkness, and the absorbance value was examined at 517 nm [3]. The results of the DPPH radical scavenging assays were demonstrated as a percentage using the following Formula (1):

% = [(1 − At/Ao)] × 100(1)

At = absorbance of MCG source, Ao = absorbance of control

The ABTS radical scavenging capacity was also evaluated following the method explained by Lee et al. [6]. For the ABTS assay, the reaction solution was composed of 7 mM ABTS (dissolved in ethanol) and 2.45 mM potassium persulfate. The above mixture was kept in the dark for 12 h at 25 °C. The ABTS solution (0.9 mL) was added to the MCG sample (0.1 mL), and the absorbance value at 734 nm was recorded. The sample or positive control (Trolox) concentrations were measured as being similar to those of the DPPH radical, and its effect was expressed as a percentage with the following Equation (2):

% = [(1 − At/Ao)] × 100(2)

At = absorbance of MCG source, Ao = absorbance of control

The FRAP value was determined according to the method described previously [23]. The FRAP solution was prepared by mixing acetate buffer (300 mM, pH 3.6), TPTZ (10 mM, in 40 mM HCl), and F_2_Cl_3_ (20 mM) at a 10:1:1 ratio, and then maintained at 25 °C for 15 min. The appropriate dilution of MCG extract (0.05 mL) was added to the FRAP solution (0.95 mL), and the mixture was incubated at 37 °C for 15 min. Thereafter, the absorbance value was read at 593 nm.

### 2.7. DNA Protection Capacity

To evaluate the DNA protection rate in the 50% ethanol extracts of the MCG source, the metal-catalyzed oxidation (MCO) DNA cleavage protection method was used, as described previously by Lee et al. [25] and Rahman et al. [26]. The supercoiled plasmid DNA (pUC18 from *E. coli*) at a concentration of 50 μg/mL was diluted in phosphate-buffered saline (0.5 M, pH 7.4). The MCG extracts of five different concentrations (50, 100, 250, 500, and 1000 μg/mL, 5 μL) were added to supercoiled plasmid DNA (5 μL), dithiothreitol (3.3 mM, 5 μL), and FeCl_3_ (15.4 μM, 5 μL), and then incubated at 37 °C for 2 h. The above reaction solution (5 μL) was added with a DNA loading buffer (1 μL) and then loaded onto a 0.8% agarose gel of Tris-acetate EDTA buffer, including Tris-acetate (40 mM) and EDTA (1 mM). The DNA gel was visualized and photographed using a UV transilluminator in the Gel Doc XR system (Bio-Rad, Hercules, CA, USA) followed by electrophoresis at 85 V for 30 min. Using Image Lab, the DNA band imaging and intensity were screened, and the following formula was used to express the DNA damage inhibition rate [26]:

DNA band protection (%) = (SF DNA band intensity/pUC18 plasmid DNA band intensity) × 100(3)

### 2.8. Evaluation of Fatty Acid Contents

The fatty acid contents (6 saturated and 10 unsaturated fatty acids) were assessed using the trifluoride-catalyzed methylation technique [6]. The organ extract (50% ethanol, 2 mL) was added with 0.5 N NaOH to methanol (3 mL) and then heated at 100 °C for 10 min. The above mixed solution was maintained for 15 min at 25 °C and added with 14% boron trifluoride (2 mL) to methanol. The crude mixture was kept for 30 min at 100 °C for fatty acid methylation and then saturated with 28% NaCl (6 mL) and isooctane (2 mL). The supernatant was centrifuged for 3 min at 3000× *g* and then filtered using a membrane filter (0.45 μm, Whatman, Inc., Maidstone, VT, USA). The filtered supernatant was measured using GC, and their contents were determined in mg/100 g. The GC analysis was programmed following the previously reported data, with slight modifications [6]: flame ionization detected temperature at 200 °C; carrier gas N_2_ at 1 mL/min; injector temperature at 200 °C; and injection volume 20 μL. Moreover, the inlet and detector temperatures were set at 250 °C. The oven temperature was set at 180 °C, raised to 230 °C at a rate of 5 °C/min, and held for 10 min.

### 2.9. Evaluation of Amino Acid Contents

The 30 amino acids, including 22 non-essential and 8 essential derivatives, were measured using previous methods [6,17]. The powdered sample (1 g) was added to distilled water (4 mL) and heated at 60 °C for 1 h. After cooling to 25 °C, the reaction solution was hydrolyzed with 5 mL of 10% sulfosalicylic acid for 60 min at 60 °C. The above mixture was centrifuged for 5 min at 3000× *g*, and the supernatants were filtered using a 0.45 μm syringe filter. The amino acid content was demonstrated in mg/100 g using an amino acid analyzer (L-8900).

### 2.10. Preparation of Samples and Calibration Curves for Ginsenoside and Phenolic Phytochemical Contents

To determine the ginsenoside and phenolic phytochemical contents in MCG organs, the establishment of samples and calibration curves were established following the methods by Cho et al. [15] and Lee et al. [6], with slight modifications. The dried samples were pulverized for 3 min using a HR2960 grinder (Philips, Drachten, The Netherlands). The powdered organs (1.0 g, 60 mesh) were extracted with 50% ethanol (20 mL) for 24 h at 25 °C in a shaking incubator and then centrifuged for 5 min at 3000× *g*. The crude supernatant was filtered using a 0.45 μm syringe filter, and the solution was measured using HPLC. The calibration curves of two metabolite structures were established on nine points (1000, 500, 250, 100, 50, 10, 5, 1, and 0.5 μg/mL) of the individual standard. To obtain 1000 μg/mL, the stock solution of each component was prepared with dimethyl sulfoxide. The peak areas of the ginsenoside and phenolic phytochemical standards were integrated from the HPLC chromatograms at 203 (ginsenoside), 280 (phenolic acid), and 270 nm (flavonol). The correlation coefficient of each curve was obtained to be higher than 0.998.

### 2.11. HPLC Operation Conditions for the Quantification of Ginsenosides and Phenolic Phytochemicals

The ginsenoside and phenolic phytochemical contents were measured as reported previously, with slight modifications [6,12,14,27]. For ginsenoside evaluations, the 50% ethanol extract (20 μL) was injected onto an analytical reversed phase C18 column, and other conditions were as follows: column temperature: 25 °C; operation time: 105 min; flow rate: 1.0 mL/min; detection: 203 nm; mobile phase: elution A (H_2_O)–elution B (CH_3_CN) using the gradient program; and mobile rate: 0–10 min (19% B), 15 min (20% B), 30 min (23% B), 42 min (30% B), 75 min (35% B), 80 min (60% B), 100 min (90% B), and 105 min (100% B). The chromatographic separation of phenolic phytochemicals was also documented using the C18-RP column with a flow rate of 1.0 mL/min at 25 °C, and the remaining conditions were processed as follows: running time: 65 min; detection: 280 nm (phenolic acid) and 270 nm (flavonol); mobile phase: elution A (0.5% CH_3_COOH in H_2_O)–elution B (CH_3_OH) using gradient system; and elution rate: 0–10 min (15% B), 15 min (20% B), 20 min (25% B), 25 min (30% B), 30 min (40% B), 35 min (50% B), 40 min (55% B), 45 min (60% B), 55 min (80% B), 60 min (90% B), and 65 min (100% B).

### 2.12. Data Processing

The fatty acid, amino acid, ginsenoside, and phenolic phytochemical contents were expressed as the mean ± standard derivation (SD) of pentaplicate measurements, and their differences were analyzed using Duncan’s multiple range test, based on the 0.05 probability level, using the statistical analysis software (SAS) 9.2 PC package (SAS Institute Inc. Cary, NC, USA). The antioxidant, TPC, and TFC were also measured as the mean ± SD values of three replicates using Microsoft Excel (Microsoft 2013, Roselle, IL, USA), and differences were analyzed using Tukey’s multiple test, based on the 0.05 probability level. The results without common superscript letters were statistically different (*p* < 0.05). Heatmap and clustering analyses were performed with the mean values in the MetaboAnalyst 6.0 (http://www.metaboanalyst.ca, accessed on 5 March 2024), and hierarchical cluster analyses were carried out using the Euclidean distance algorithm. Principal cluster analysis (PCA) was conducted using the OriginLab 10.0 software (Northampton, MA, USA), generating score plots for PC1 and PC2 within 95% confidence intervals. Heatmaps were based on the Person correlation distance and Ward’s clustering measure, following the method published by Pang et al. [28].

## 3. Results

### 3.1. Weight of Organs According to Maturation Periods

The average ratios of each organ (leaves, stems, and roots) per MCG at different harvest times are shown in Figure S2. These ratios indicated the proportion of each organ to the total mass of the MCG plant. In fresh MCG, the ratio of each part slightly changed according to maturation times (17 May → 31 May → 21 June → 13 July) as follows: leaves (29.2 → 25.5 → 25.3 → 28.3%); stems (33.5 → 41.0 → 39.2 → 30.4%); and roots (37.3 → 33.5 → 35.5 → 41.3%). From 17 May to 31 May, the weight ratio shifted from leaves and roots to stems. Conversely, from 31 May to 13 July, the weight was redistributed from stems back to leaves and roots. The dried MCG data showed that the weight ratios of the leaves (25.8 → 21.7 → 18.7 → 19.3%) and stems (24.4 → 25.1 → 23.9 → 20.4%) decreased, while the ratio of roots per MCG (49.8 → 53.2 → 57.4 → 60.3%) increased.

### 3.2. Comparisons of TPC and TFC in Mountain-Cultivated Ginseng Organs through Different Maturation Periods

**Figure 1:**
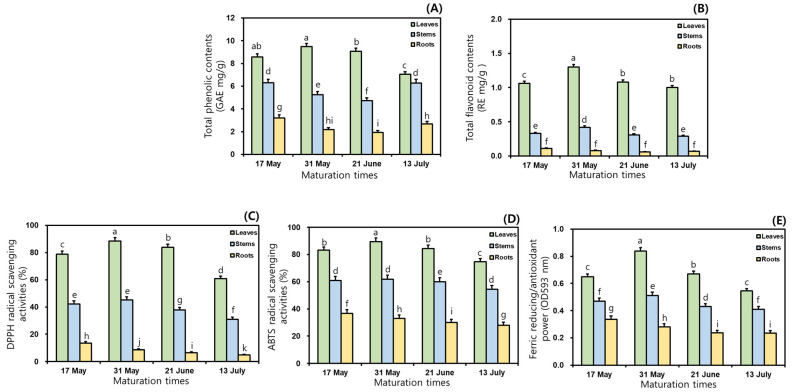
Comparisons of TPC, TFC, and antioxidant properties in the 50% ethanol extracts of MCG organs at maturation times: (**A**) TPC; (**B**) TFC; (**C**) DPPH radical scavenging activities; (**D**) ABTS radical scavenging activities; and (**E**) FRAP. Data are expressed as the mean ± SD, and differences were analyzed using Tukey’s test, *n* = 3. The results without common superscript letters (a–k) were statistically different (*p* < 0.05).

This study examined the TPC and TFC in the 50% ethanol extracts of MCG organs at different maturation times. As shown in Figure 1, the TPC and TFC ratios varied considerably between maturation periods and organs; specifically, significant differences in TPC were observed among the three organs. Regarding TPC, the MCG leaves exhibited the most abundant in all maturation times, followed by stems and roots, and their average ratios were recorded as follows: 8.55 (leaves) > 5.64 (stems) > 2.50 GAE mg/g (roots) (Figure 1A). The leaves on 31 May exhibited the highest TPC value with 9.48 GAE mg/g, whereas the roots collected on 21 June (1.93 GAE mg/g) had the lowest ratio (Figure 1A). The rank order of the remaining samples was as follows: leaves at 21 June (9.07 GAE mg/g) > leaves at 17 May (8.58 GAE mg/g) > leaves at 13 July (7.05 GAE mg/g) > stems at 17 May (6.30 GAE mg/g) > stems at 13 July (6.28 GAE mg/g) > stems at 31 May (5.25 GAE mg/g) > stems at 21 June (4.73 GAE mg/g) > roots at 17 May (3.21 GAE mg/g) > roots at 13 July (2.69 GAE mg/g) > roots at 31 May (2.17 GAE mg/g) (Figure 1A). In the TFC analysis, when the MCG were grown for longer times in the range of 17 May → 13 July, the distributions of the average TFC occurred in the following order: leaves (1.11 RE mg/g) > stems (0.34 RE mg/g) > roots (0.08 RE mg/g) (Figure 1B). All collected leaves displayed predominant TFC ratios, compared to the patterns in other organs. The highest value of 1.30 RE mg/g was observed in leaves harvested on 31 May, followed by 21 June (1.08 RE mg/g) > 17 May (1.06 RE mg/g) > 13 July (1.00 RE mg/g) (Figure 1B). The MCG stems and roots exhibited TFC values in increasing order: stems: 0.42 RE mg/g (31 May) > 0.33 RE mg/g (17 May) > 0.31 RE mg/g (21 June) > 0.29 RE mg/g (13 July); roots: 0.11 RE mg/g (17 May) > 0.08 RE mg/g (31 May) > 0.07 RE mg/g (13 July) > 0.06 RE mg/g (21 June) (Figure 1B).

### 3.3. Variations of Antioxidant Effects in Mountain-Cultivated Ginseng Organs through Different Maturation Times

In this study, in vitro techniques such as the radical scavenging assay and FRAP method were used because of their stability, reproducibility, simple control, and cost effectiveness with spectrophotometry [23,25,29,30]. To evaluate antioxidant effects, DPPH and ABTS assays were performed according to the percentage inhibition of radical formation by comparing positive controls of BHT and Trolox [25,31]. In the preceding tests, the scavenging abilities of the sample and positive control against DPPH radical increased with increasing concentrations through 250 → 500 → 1000 → 2000 → 4000 μg/mL. Although the 50% ethanol extracts were observed to have 100% scavenging capacities at 1000, 2000, and 4000 μg/mL, this study was conducted to investigate the radical scavenging properties at 500 μg/mL because of the dose-dependent changes in the inhibition ratios. This result was similar to the earlier study concerning processed MCG products through aging and fermentation [15]. As presented in Figure 1C, the inhibition percentages on DPPH radical exhibited remarkable differences in MCG organs, compared to those in maturation times. The average DPPH radical inhibition effects in three organs were ranked as follows: leaves (77.9%) > stems (39.1%) > roots (8.4%). The highest scavenging ability against this radical accounted for 88.4% in the leaves of 31 May, while the roots at 13 July displayed the lowest effect (4.9%) (Figure 1C). Specifically, the most predominant activity was shown in the MCG leaves of the 31 May with 88.4%, and the rates of the remaining leaves were as follows: 21 June (83.8%) > 17 May (78.8%) > 13 July (60.9%). For these above reasons, the MCG leaves on the harvested sample of 31 May displayed the best excellent DPPH radical scavenging effect at 500 μg/mL. The MCG stems and roots showed slight differences when compared to those of leaves in the following order: stems: 31 May (45.2%) > 17 May (42.3%) > 21 June (37.8%) > 13 July (31.0%); roots: 17 May (13.4%) > 31 May (8.6%) > 21 June (6.6%) > 13 July (4.9%) (Figure 1C). Especially the antioxidant abilities against DPPH radical may be associated with the high abundance of phenolics, including quercetin, catechin, epicatechin, and chlorogenic acid (Section 3.7). In the ABTS radical method, the 50% ethanol extracts of MCG organs showed similar patterns to the DPPH radical inhibition results. The scavenging capacities on this radical displayed extremely considerable differences in organs from their effects through maturation times; specifically, all extracts had higher ABTS scavenging activities when compared to the DPPH radical (Figure 1D). During four maturation times, the average ABTS radical scavenging abilities occurred as follows: leaves (83.0%) > stems (59.3%) > roots (31.9%). As mentioned for the DPPH radical inhibitions, the MCG leaves exhibited the highest ABTS scavenging effects, and especially the collected leaves on 31 May displayed an absolutely dominant ability with 89.5%. Although this source was observed to have a low ABTS-scavenging capacity when compared to the Trolox (positive control, 91.0%), the collected leaves on 31 May may be recommended as a potential candidate for increasing MCG value in the development of human health-promoting agents. The remaining leaves also showed high inhibition ratios as follows: 21 June (84.5%) > 17 May (83.1%) > 13 July (74.8%). The ABTS radical scavenging effects of the stems and roots were ranked as follows: stems: 31 May (61.8%) > 17 May (60.9%) > 21 June (60.0%) > 13 July (54.5%); roots: 17 May (36.6%) > 31 May (32.9%) > 21 June (30.2%) > 13 July (27.9%) (Figure 1D). The FRAP patterns were similar to the results obtained using the radical inhibitions; specifically, their antioxidant orders through organs and maturation times were consistent with those of the ABTS radical scavenging capacities (Figure 1E). The average FRAP values exhibited the highest activity in leaves with 0.68 OD_593 nm_, and other organs displayed a decreasing order: stems (0.46 OD_593 nm_) > roots (0.27 OD_593 nm_). In addition, the MCG leaves were ranked at four maturation times as follows: 0.84 (31 May) > 0.67 (21 June) > 0.65 (17 May) > 0.55 OD_593 nm_ (13 July) (Figure 1E). The FRAP values of the stems and roots showed the same tendencies, with mild differences: stems: 0.51 (31 May) > 0.47 (17 May) > 0.43 (21 June) > 0.41 OD_593 nm_ (13 July); roots: 0.34 (17 May) > 0.28 (31 May) > 0.24 (21 June) > 0.23 OD_593 nm_ (13 July).

### 3.4. Comparison of DNA Protection Properties in Mountain-Cultivated Ginseng Organs through Different Maturation Times

**Figure 2:**
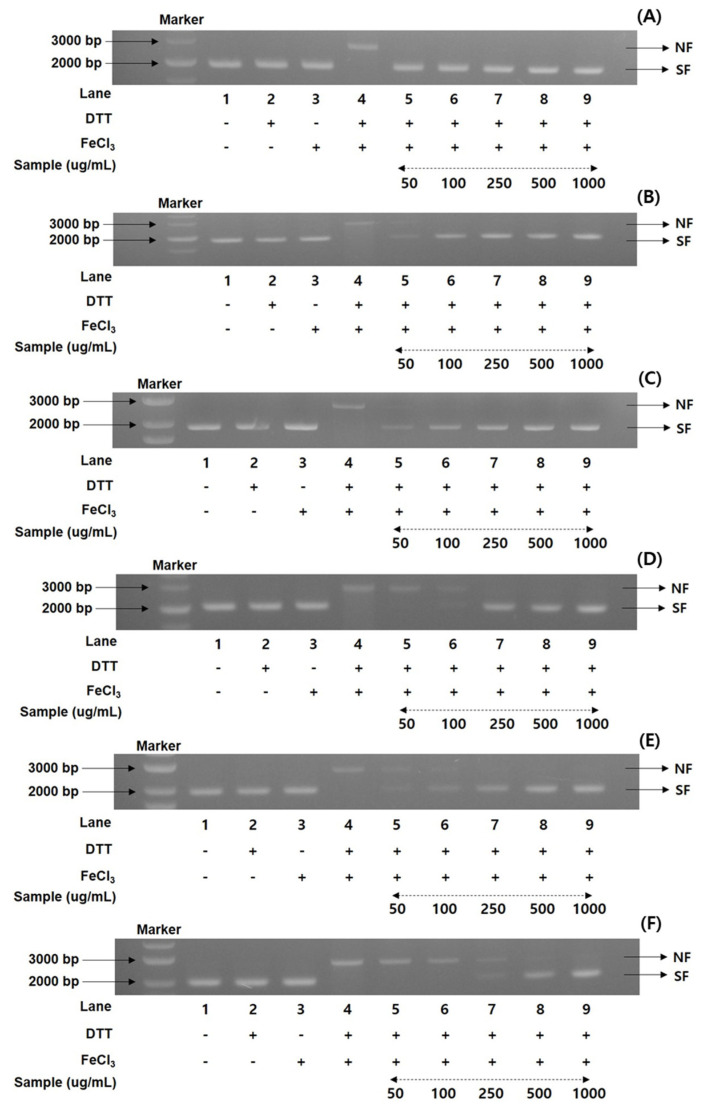
Comparison of DNA protectant properties in the 50% ethanol extracts of MCG organs at maturation times: (**A**) DNA protectant effects of leaves on 31 May; (**B**) DNA protectant effects of leaves on 13 July; (**C**) DNA protectant effects of stems on 31 May; (**D**) DNA protectant effects of stems on 13 July; (**E**) DNA protectant effects of roots on 17 May; and (**F**) DNA protectant effects of roots on 31 July. Lane 1, pUC18 only; lane 2, pUC18 with DDT only; lane 3, pUC18 with FeCl_3_ only; lane 4, pUC18 with MCO system; lane 5–9, pUC18 with combinant extracts in the MCO system (lane 5: 50 μg/mL, lane 6: 100 μg/mL, lane 7: 250 μg/mL, lane 8: 500 μg/mL, and lane 9: 1000 μg/mL).

To gain more information on the antioxidant ratios of the MCG plant, the DNA protection activities of recombinant 50% ethanol extracts from the organs through maturation times were evaluated. A nicked DNA system through super-coiled plasmid DNA pUC18 was investigated in an MCO apparatus [25,26,32]. According to the preliminary results of the radical scavenging effects and FRAP values, the DNA protection assay documented the percentage values in five different concentrations (50, 100, 250, 500, and 1000 μg/mL). Using gel electrophoresis, the MCG extracts were measured for their DNA damage protection abilities on the hydroxyl radical produced with the nicked DNA pUC18. As illustrated in Figure 2, the DNA protection rates were significantly different in each extract of organs and maturation times.

Their patterns were consistent with the antioxidant properties of radical scavenging effects and FRAP ratios as follows: 31 May > 21 June > 17 May > 13 July, with the order of leaves > stems > roots. Although the DNA protection rates exhibited remarkable differences in the various concentrations between organs and maturation times, the present results showed the highest and lowest protection bands in each organ (Figure 2). The collected leaves on 31 May displayed the highest protection rates (100% in all concentrations) in diverse concentrations in the range of 50–1000 μg/mL, based on the DNA marker (pUC18 only, control) (Figure 2A). The DNA band patterns of the lowest protection ratios in the leaves on 13 July were detected with 86.2, 95.4, and 100% at 50, 100, and 250 μg/mL (Figure 2B), respectively, and the remaining concentrations (500 and 1000 μg/mL) showed 100% DNA protection patterns. In the MCG stems, the highest DNA protection effects were displayed in the sample collected on 31 May (Figure 2C), while the collected sample on 13 July exhibited the lowest protection patterns (Figure 2D). More specifically, at a concentration 50 μg/mL, these two extracts preserved the mobility of DNA fragments by 73.3 and 67.5%, respectively, compared to the control. The DNA protection efficiency at other concentrations slightly increased, with an increase in concentration (100 → 1000 μg/mL) as follows: 31 May: 88.9 100 → 100 → 100%; 13 July: 79.3 → 91.4 → 100 → 100% (Figure 2C,D). The MCG roots also exhibited similar patterns to those of leaves and stems. However, their DNA protection rates showed lower values than other organs, with considerable differences in the treatment concentrations: roots on 17 May: 63.9 → 73.6 → 85.5 → 93.9 → 100%; roots on 13 July: 60.4 → 62.2 → 75.9 → 85.7 → 100%, in accordance with the increase in patterns of 50 → 1000 μg/mL (Figure 2E,F).

### 3.5. Changes in Fatty Acid Contents in Three Organs of Mountain-Cultivated Ginseng during Different Maturation Times

The fatty acid contents (6 saturated and 10 unsaturated fatty acids) in each organ of MCG at maturation times are shown in Table 1. The leaves, stems, and roots of the MCG plant showed considerable differences in total fatty acids in the ranges of 831.2–1057.9, 635.3–847.1, and 415.0–954.9 mg/100 g. Their average contents were in the following order: leaves (892.0 mg/100 g) > stems (728.8 mg/100 g) > roots (674.1 mg/100 g). Furthermore, significant differences in total fatty acids were detected in the maturation times of individual organs, as follows: leaves (891.7 → 1057.9 → 787.3 → 831.2 mg/100 g), stems (635.3 → 740.2 → 847.1 → 692.5 mg/100 g), and roots (954.9 → 888.3 → 438.3 → 415.0 mg/100 g) (Table 1). The highest fatty acid contents for each organ were observed as follows: leaves on 31 May (1057.9 mg/100 g), stems on 21 June (847.1 mg/100 g), and roots on 17 May (954.9 mg/100 g). Conversely, the lowest contents were found in leaves on 13 July (831.2 mg/100 g), stems on 17 May (635.3 mg/100 g), and roots on 13 July (415.0 mg/100 g), respectively. Additionally, the fatty acid contents in the leaves and roots collected on 17 May and 31 May were higher than those in the stems, and the total content in the leaves and stems on 21 June and 13 July showed higher ratios than the roots (Table 1). Extending the maturation times from 17 May to 31 May markedly increased the total fatty acid content in leaves and stems, while the MCG organs at other maturation times exhibited diverse patterns (Table 1). Specifically, when MCG leaves were allowed to grow for a longer duration (17 May → 31 May), the total fatty acid content increased (891.7 → 1057.9 mg/100 g). However, for the period extending from 31 May to 21 June, there was a decrease in total fatty acid content from 1057.9 to 787.3 mg/100 g. In the harvested stems on 17 May → 21 June, the total fatty acids showed an increasing trend (635.3 → 740.2 → 847.1 mg/100 g), and the MCG roots decreased markedly with 954.9 → 888.3 → 438.3 → 415.0 mg/100 g, depending on the maturation times (17 May → 13 July). Among the various compositions, palmitic acid and linoleic acid exhibited the most abundant contents in saturated and unsaturated fatty acids, representing approximately 60% of the total average content.

The contents of the above components were high, with remarkable differences in all maturation times and MCG in the ranges of 100.2–242.9 (palmitic acid) and 188.4–476.1 (linoleic acid) mg/100 g. Furthermore, two compositions showed the predominant average contents with 224.7 (25.2%), 161.8 (22.2%), and 159.5 (23.7%) mg/100 g (palmitic acid), as well as 247.8 (27.8%), 342.8 (47.0%), and 324.4 (48.1%) mg/100 g (linoleic acid) in leaves (average 892.0 mg/100 g), stems (average 728.8 mg/100 g), and roots (average 674.1 mg/100 g). In addition, α-linolenic acid content was the highest in leaves, changing from 294.0 mg/100 g (17 May) to 345.2 mg/100 g (31 May), then decreasing to 126.3 mg/100 g (21 June) and finally to 81.8 mg/100 g (13 July), compared with other organs (Table 1). In the remaining fatty acids, oleic acid content ranged from 28.8 to 188.5 mg/100 g (average: 88.8 mg/100 g), and other compositions showed minor amounts (˂10 mg/100 g), with slight variations.

### 3.6. Changes in Amino Acid Contents in Three Organs of Mountain-Cultivated Ginseng during Different Maturation Times

Although amino acids have excellent functional values in crops and natural sources, to our knowledge, no studies have reported the variations of their contents and compositions in MCG plants at maturation times. As indicated in Table 2, the 30 amino acid components, including 22 non-essential and 8 essential derivatives, were investigated in MCG organs according to their maturation times. The non-essential amino acids had an average content of 545.8 mg/100 g, which is approximately two-fold that of the essential amino acids (249.2 mg/100 g), as detailed in Table 2. Moreover, individual and total amino acid contents exhibited remarkable variations in maturation times and organs. During the maturation times from 17 May to 13 June, the amino acid content in MCG leaves increased remarkably (965.2 → 1153.3 → 1182.5 → 1989.2 mg/100 g), whereas those of the roots and stems showed decreasing trends, with contents changing between 1027.9 → 532.4 → 406.5 → 382.8 and 666.5 → 476.1 → 397.7 → 359.5 mg/100 g (Table 2). The MCG leaves showed the highest average content (1322.6 mg/100 g), and the contents of the remaining organs were in the following order: roots (587.4 mg/100 g) > stems (475.0 mg/100 g). In summary, MCG leaves displayed the most substantial content of amino acids compared to other organs; specifically, the harvested sample on 13 July had the highest total content (1989.2 mg/100 g). Specifically, the GABA contents in all the collected leaves had high ratios of 106.7 (17 May) → 158.0 (31 May) → 181.5 (21 June) → 211.7 mg/100 g (13 July) compared to other amino acids, with alanine levels following this pattern: 84.1 → 125.3 → 128.6 → 100.0 mg/100 g (Table 2). Furthermore, the arginine content showed decreasing patterns, with mild variations of 79.4 → 37.5 → 33.9 → 50.6 mg/100 g, while ornithine did not show remarkable differences (16.2 → 11.2 → 4.4 → 5.0 mg/100 g). The arginine ratios of the predominant content in MCG roots decreased significantly, showing considerable variations: 382.4 → 194.0 → 140.0 → 50.6 mg/100 g, compared with other compositions (Table 2). In particular, the contents of three amino acids (aspartic acid, aspartic acid-NH_2_, and glutamic acid) in leaves increased rapidly by approximately 3–9 times, 49.7 → 177.0, 34.3 → 324.9, and 40.6 → 118.9 mg/100 g at different maturation times, and the valine, isoleucine, leucine, and phenylalanine contents exhibited highly increased rates of 78.2 → 136.0, 65.3 → 105.3, 80.3 → 132.8, and 73.0 → 127.6 mg/100 g, respectively (Table 2). Overall, the total amino acid content in MCG leaves increased (1182.5 → 1989.2 mg/100 g; non-essential: 735.8 → 1293.9 and essential: 446.7 → 695.3 mg/100 g), mainly with high variations, and their ratios were increased approximately two times. Although the change was not large in stems (397.7 → 359.5 mg/100 g; non-essential: 269.7 → 254.7 and essential: 128.0 → 104.8 mg/100 g) and roots (406.5 → 382.8 mg/100 g; non-essential: 315.4 → 295.5 and essential: 91.1 → 87.3 mg/100 g), the content of essential and non-essential amino acids and total amino acids decreased.

### 3.7. Fluctuation of Ginsenosides in Mountain-Cultivated Ginseng Organs during Different Maturation Times

**Figure 3:**
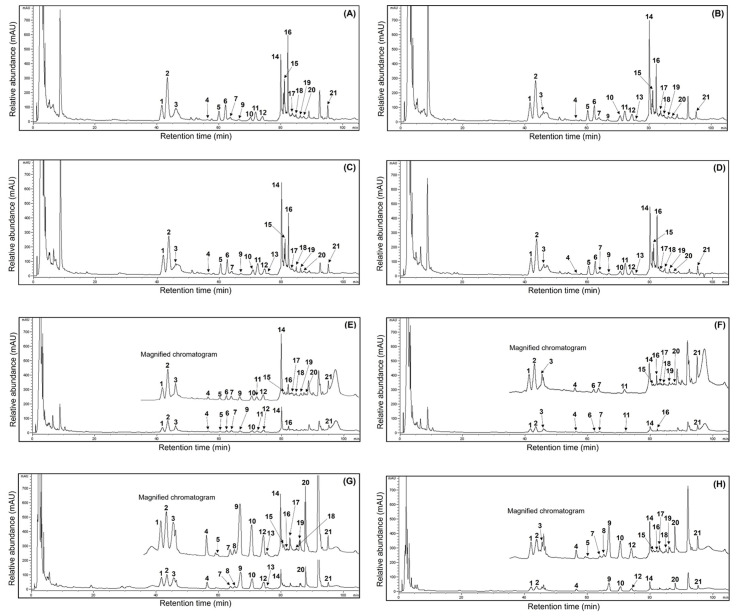
Comparison of HPLC chromatograms of 21 ginsenosides from the 50% ethanol extracts of MCG organs at different maturation times: (**A**) leaves (17 May); (**B**) leaves (31 May); (**C**) leaves (21 June); (**D**) leaves (13 July); (**E**) stems (31 May); (**F**) stems (17 May); (**G**) roots (17 May); and (**H**) roots (21 June). peak 1, Rg1; peak 2, Re; peak 3, Ro; peak 4, Rf; peak 5, F5; peak 6, F3; peak 7, Rg2; peak 8, Rh1; peak 9, Rb1; peak 10, Rc; peak 11, F1; peak 12, Rb2; peak 13, Rb3; peak 14, Rd; peak 15, Rd2; peak 16, F2; peak 17, Rg3; peak 18, protopanaxtriol; peak 19, compound K; peak 20, Rh2; peak 21, protopanaxdiol.

Much valuable research has recently focused on the ginsenoside profiles and their amounts in ginseng sources and processed foods because of their human health benefits [1,3,15,16,19,21,22]. Unfortunately, few reports have demonstrated fluctuations in the ginsenoside content of ginseng sources through maturation times. Thus, this study analyzed the ginsenoside compositions in MCG organs during maturation times. The presentative HPLC chromatogram of ginsenoside peaks is shown in Figure 3, and the ginsenoside contents were measured by comparing their retention times using ginsenoside standards in the HPLC analysis [6,15]. The 21 ginsenoside components, including 9 protopanaxtriol, 11 protopanaxdiol, and 1 oleanane derivatives, are summarized in Table 3. The individual and total ginsenosides showed remarkable differences between organs and maturation times; especially the MCG leaves displayed high ginsenoside content. In other words, the leaves were detected to have the predominant average content of 83.5 mg/g at four maturation times (Figure 3A–D), and the content in the remaining organs exhibited the following order: roots (30.1 mg/g) > stems (16.5 mg/g). The most abundant total ginsenosides were detected in the 31 May sample (147.6 mg/g), showing significant differences in each organ (leaves: 88.7 mg/g; stems: 23.0 mg/g; roots: 35.9 mg/g), followed by 17 May (133.8 mg/g) > 13 July (122.3 mg/g) > 21 June (116.6 mg/g). In particular, the MCG leaves on 17 May showed the highest ginsenoside ratios (84.8 mg/g) (Figure 3A) compared with those in stems (8.2 mg/g) (Figure 3F) and roots (40.8 mg/g) (Figure 3G). Also, ginsenosides Re and F2 showed high distributions with 19.1 and 13.1 mg/g, representing approximately 22.5 and 15.5% of the total content (84.8 mg/g), respectively, followed by ginsenoside Rd (8.5 mg/g at 10.0%) > Ro (9.8 mg/g at 11.6%) > Rd2 (8.4 mg/g at 10.0%) > F3 (7.9 mg/g at 9.3%) > Rg1 (4.5 mg/g at 5.3%) > F1 (2.2 mg/g at 2.6%) (Table 3). Compared to other maturation periods, the 17 May leaves demonstrated higher ratios in fresh and dry weight, indicating an association with leaf maturation and ginsenoside content. Even though the distributions of PPT, PPD, and oleanane structures were 37.8, 37.2, and 9.8 mg/g in the current results, these data demonstrated significant variations compared to earlier reports, which revealed that the PPT type showed large content in other ginseng sources [5,6]. Their distributions in the stems and roots on 17 May were as follows: stems: PPT (4.5 mg/g) > PPD (2.8 mg/g) > oleanane (0.9 mg/g); roots: PPD (22.5 mg/g) > PPT (11.6 mg/g) > oleanane (3.7 mg/g) (Table 3). Moreover, their ratios showed similar patterns in the organs of all harvested samples. The MCG stems detected small ginsenoside amounts, with mild variations in the range of 8.2–23.0 mg/g during the four maturation times (Figure 3E,F). Interestingly, the total ginsenosides of the PPD type in all MCG roots exhibited approximately three-fold higher content (Figure 3G,H) than PPT compared to those in other organs (PPD:PPT ratio; leaves: 1:1, stems: 1:1) (Table 3). Extending the maturation periods between the 31 May and 13 July samples showed remarkable variation in ginsenoside accumulations, similar to the patterns of ginsenoside from the 17 May source. Among diverse compositions, the contents of ginsenosides Re, Rd, F2, and F3 had high average ratios of 17.9, 11.3, 10.9, and 7.6 mg/g, respectively, compared to other components in all MCG leaves (Figure 3A–D and Table 3). The above components also showed significant rates, with high content in the following order in the leaves during 31 May → 13 July: ginsenoside Re, 18.2 → 17.3 → 16.9 mg/g; F3, 7.9 → 7.6 → 6.9 mg/g; Rd, 14.8 → 13.0 → 8.7 mg/g; and F2, 10.1 → 10.8 → 9.5 mg/g. In all MCG roots, ginsenoside Rb1 exhibited the highest content in the range of 4.3–10.3 mg/g; especially the harvested samples on 17 May and 31 May exhibited the highest ratios, with 10.3 (25.2%) and 9.5 mg/g (26.5%) of the total ginsenoside content (40.8 and 35.9 mg/g) (Figure 3G,H). The second main compound was ginsenoside Re (average content: 4.0 mg/g) with 5.7, 4.2, 2.6, and 3.3 mg/g, and other derivatives displayed the following order: Rc > Rb2 > Rd, with average contents of 3.1, 2.5, and 2.1 mg/g, respectively (Table 3). In MCG stems, ginsenoside Re was the most abundant component (average content: 3.9 mg/g) observed at levels of 2.3, 4.9, 4.8, and 3.6 mg/g, and ginsenoside Rd (average content: 2.5 mg/g) had the second compound showing mild variations with levels at 1.3, 4.0, 2.8, and 1.8 mg/g, respectively (Figure 3E,F). Other ginsenosides were present in low concentrations (averaging, <1.0 mg/g), except for Rg1, which had an average content of 1.2 mg/g (Table 3). In summary, the MCG leaves contained ginsenosides that were approximately 2.5 and 5 times higher, with an average of 83.5 mg/g, than the roots (30.1 mg/g) and stems (16.5 mg/g) during 17 May → 13 July (Figure 3). Furthermore, the ginsenosides Rd, F2, and F3 of leaves had high average contents of 11.3, 10.9, and 7.6 mg/g, respectively. The predominant average ginsenoside in each organ exhibited significant differences, with the increased rates as follows: ginsenoside Re (17.9 mg/g, leaves) > ginsenoside Rb1 (7.9 mg/g, roots) > ginsenoside Re (3.9 mg/g, stems).

### 3.8. Fluctuation of Phenolic Phytochemicals in Mountain-Cultivated Ginseng Organs during Different Maturation Times

Numerous studies have confirmed that major metabolites, such as ginsenosides in ginseng, play significant roles in the development of functional foods and nutraceuticals that serve as health-promoting agents for humans [1,4,6,12,13]. However, little data have demonstrated the comparison and quantification of phenolic phytochemicals in ginseng tissues during maturation times. For this reason, we designed to measure the phenolic compounds in the 50% ethanol extracts of MCG organs at different maturation times. As illustrated in Table 4, although the individual contents displayed remarkable differences between organs and maturation times, the total phenolics showed slight variations, ranging from 973.1 to 1126.5 μg/g. To be more specific, the highest total phenolic content of 1126.5 μg/g was detected in the 31 May sample, followed by the 17 May (1036.3 μg/g) > 21 June (1009.2 μg/g) > 13 July (973.1 μg/g) samples. Moreover, the MCG leaves possessed high phenolic levels, which vary in concentration when compared to the stems and roots. In the 17 May sample, the MCG leaves exhibited a high phenolic content of 524.8 μg/g (phenolic acid: 114.4 μg/g and flavonol: 410.4 μg/g) compared to other organs (stems: 302.8 μg/g; roots: 208.7 μg/g), and the flavonol derivatives were observed to have approximately four times higher content than phenolic acids. These observations showed the same patterns in all the collected MCG samples. Especially catechin and quercetin exhibited the predominant content with 125.5 and 131.7 μg/g, representing about 23.9% and 25.0% of the total content (524.8 μg/g), and the remaining phenolics were present in the following order: chlorogenic acid (57.9 μg/g, 11.0%) > epigallocatechin (52.2 μg/g, 9.9%) > epicatechin (48.9 μg/g, 9.3%) > 4-hydroxylbenzoic acid (36.0 μg/g, 6.9%) > other phenolics (<20%) (Table 4). When the MCG plant is grown for longer times in the range 31 May → 13 July, the individual and total phenolic contents showed similar patterns compared to those in the 17 May sample. The phenolic content in the leaves, stems, and roots consistently decreased, and their decreased rates were ranked as follows: leaves: 638.3 → 547.8 → 510.1 μg/g > stems: 309.8 → 296.6 → 285.5 μg/g > roots: 178.4 → 164.8 → 177.5 μg/g (Table 4). Interestingly, all harvested leaves in the above periods exhibited high content in catechin and quercetin with 99.4 → 119.2 → 102.9 μg/g and 197.9 → 177.7 → 172.6 μg/g, as evidenced by the result of the 17 May sample. Most of the remaining phenolic content also exhibited similar patterns. The MCG stems exhibited the predominant composition in quercetin with 100.9 (17 May, 33.3% in 302.8 μg/g) → 113.4 (31 May, 36.6% in 309.8 μg/g) → 119.1 (21 June, 40.4% in 309.8 μg/g) → 128.7 (13 July, 45.1% in 285.5 μg/g), and the second main composition was observed with concentrations of 51.5 → 90.6 → 56.2 → 31.7 μg/g in epigallocatechin (average content: 57.5 μg/g) (Table 4).

Other phenolic content are displayed in the following order: naringenin (average content: 21.9 μg/g) > catechin (average content: 20.5 μg/g) > chlorogenic acid (average content: 19.0 μg/g) > *p*-hydroxylbezoic acid (average content: 12.2 μg/g), and the remaining phenolics had low contents (<10 μg/g). The accumulation of phenolics in MCG roots showed similar tendencies as those observed in the leaves and stems. Quercetin had the highest content, displaying variations across four maturation periods: 82.4 → 107.9 → 99.1 → 99.7 μg/g, with an average content of 97.3 μg/g (accounting for 53.3%), followed by epigallocatechin (average 28.0 μg/g, 15.4%) > chlorogenic acid (average 14.3 μg/g, 7.8%), and other phenolics were found to be low, averaging less than <10 μg/g (Table 4).

### 3.9. Correlation Analysis of Metabolite Constituents in Three Mountain-Cultivated Ginseng Organs at Four Maturation Times

**Figure 4:**
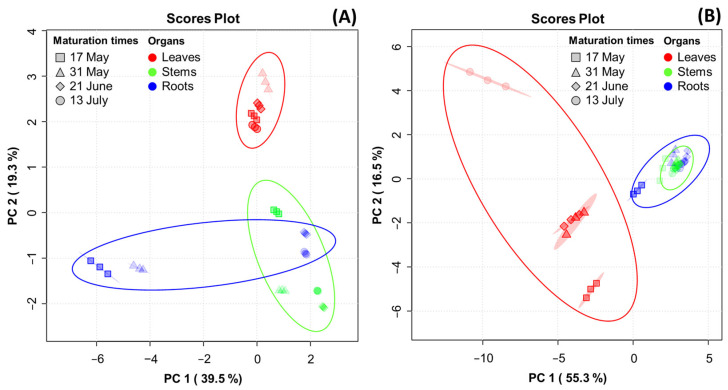

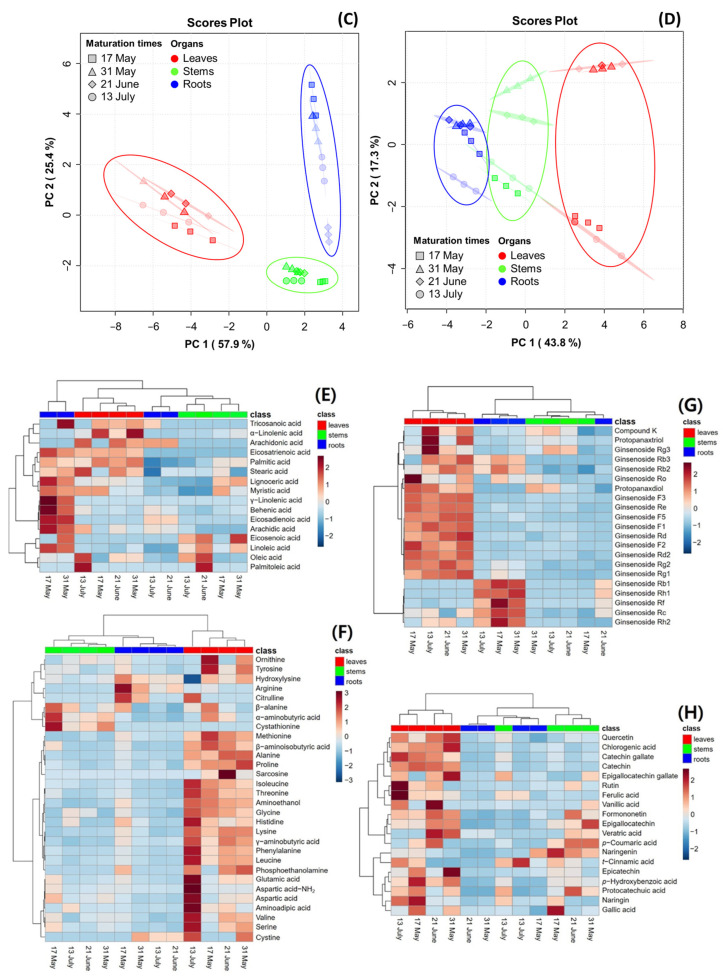
Score plot of principal component analysis (PCA) and heatmap analysis of metabolites in MCG organs at different maturation times. (**A**–**D**) Score plot of PCA of four metabolites: (**A**) fatty acids; (**B**) amino acids; (**C**) ginsenosides; and (**D**) phenolic phytochemicals. (**E**–**H**) Hierarchical clustering and heatmap analysis to investigate the variable metabolites’ relationships, either positive (red) or negative (blue): (**E**) fatty acids; (**F**) amino acids; (**G**) ginsenosides; and (**H**) phenolic phytochemicals. The mean values of the various conditions were normalized and clustered in the heatmap. The color displays the intensity of the normalized mean values of different parameters. A value of *p* < 0.05 was used to determine statistically significant difference.

PCA was performed to reveal the association of metabolite constituents (fatty acids, amino acids, ginsenosides, and phenolic phytochemicals) through maturation times in the organs of the MCG plant. Figure 4 presents the PCA score plot, which is based on the metabolite constituents of three organs at four maturation times. The percentages representing the variability explained by the PC1 and PC2 axes for each metabolite were as follows: fatty acids (58.8%) (Figure 4A), amino acids (71.8%) (Figure 4B), ginsenosides (83.3%) (Figure 4C), and phenolic phytochemicals (61.1%) (Figure 4D). A heatmap was used for the quantitative analysis of metabolite constituents using a z-score, revealing the hierarchical clustering of metabolites within each sample, as well as their positive (red) or negative (blue) association (Figure 4E–H).

In the fatty acid analysis, tricosanoic acid, α-linolenic acid, and arachidonic acid were found to be highly significant in MCG leaves at all maturation times. The MCG roots exhibited high relevance for all metabolites in the harvested samples on 17 May and 31 May, whereas a decrease in significance was observed in samples on 21 June and 13 July. The collected stems also showed high relevance for eicosenoic acid and linoleic acid. Specifically, the unsaturated fatty acid components, oleic acid and palmitoleic acid, were significantly associated with the leaves on 13 July and stems on 21 June (Figure 4E). In the amino acid profile, a high correlation was observed among most components in the MCG leaves (Figure 4F). Notably, the levels of ornithine, tyrosine, and methionine decreased, while glutamic acid, aspartic acid-NH_2_, aspartic acid, and γ-aminobutyric acid increased according to the maturation times. Moreover, the MCG stems on 17 May exhibited high relevance for β-alanine, α-aminobutyric acid, and cystathionine, while the roots were highly relevant for hydroxylysine, arginine, and citrulline (Figure 4F). However, as the maturation time progressed, the relevance of the above components tended to decrease. Regarding ginsenosides, the MCG leaves were highly associated with ginsenosides F1, F2, F3, F5, Rd, Rd2, Re, Rg2, and PPD, while the roots demonstrated relevance to ginsenosides Rb1, Rf, Rh1, and Rh2 (Figure 4G). Most phenolic phytochemicals showed a strong correlation in the MCG leaves, with significant variations depending on the maturation times (Figure 4H). Additionally, the major components such as epigallocatechin, *p*-coumaric acid, and naringenin were found in the stems at all maturation times except 13 July, and the MCG roots showed low relevance in all phenolic phytochemicals (Figure 4H).

## 4. Discussion

To the best of our knowledge, there are few reports investigating the fluctuation of the metabolite constituents and antioxidant properties in MCG organs at maturation times. However, many studies have reported that TPC and TFC ratios are associated with various biological concerns to human health [3,24,27]. The above two characteristic values were attributed to secondary metabolite contents, such as phenolic and flavonoid derivatives [23,26,33,34]. As observed in other crops, our findings may be significantly influenced by the variations in phenolic content through different organs and maturation times in MCG [16,25,27,31,35,36]. The TPC values are responsible for the highest antioxidant effects of leaves (Section 3.3) and MCG organs. This source may be an important natural antioxidant agent for functional foods. Our results are similar to those in the earlier reported literature on ginseng leaves [3,16,37], and phenolic metabolite accumulations in MCG organs may not be connected with growth times through maturity ratios [31,36]. Overall, the TPC and TFC values might positively correlate with environmental factors, including plant organs, harvest times, growth stages, and genetics [16,27,31,34]. In particular, the decrease in TPC and TFC in leaves harvested from 31 May to 13 July may be influenced by the ratios of phenolic metabolite accumulations or conversions through the biosynthetic pathways of the MCG plant during maturation [16,17,38]. Furthermore, their differences may be associated with the finding that antioxidant properties were positively related to the cellular division, biosynthesis, and transport of phenolics in the maturity periods and organs of plants [35,36]. Considering the TPC and TFC, MCG leaves may be considered an effective source for developing nutraceuticals and functional agents because of their high phenolic ratios. Our suggestion is that the ideal harvesting time for MCG leaves to achieve maximum phenolics might be that of the 31 May sample.

The differences of antioxidant properties may positively correlate with diverse secondary metabolites, including phenolics and flavonoids in each organ [26,27,34,35]. In other words, the DPPH radical inhibition ratios could be significantly influenced by phenolic phytochemicals and other metabolites in MCG samples, as well as environmental factors (organ, temperature, moisture, light, etc.) through the maturation times [3,16,37]. The results of this study, with the aim of developing new antioxidants, indicate that the optimal MCG sample was harvested on 31 May in a five-year-old source. Even though all MCG sources were observed to have lower scavenging capacities when compared to the positive control (BHT, 91% at 500 μg/mL), the MCG leaves on 31 May can be considered to be a potentially important source of natural antioxidants based on the TPC and TFC as well as phenolic phytochemicals (Section 3.1 and Section 3.7) [3,37]. Our results may be affected by the fact that the DPPH radical is associated with scavenging properties through the hydrogen-donating effects of various phenolics in the 50% ethanol extracts of MCG samples, and the ABTS radical is crucial for evaluating hydrogen-donating and chain-breaking activities, as indicated in the previous literature [6,29,31]. These distributions may be influenced by the accumulated states of metabolites in the organs during growth periods [10,19,27,36]. Overall, the inhibition patterns against ABTS radical exhibited higher properties with approximately 5, 15, and 20% in leaves, stems, and roots, respectively, when compared to the DPPH radical results. Furthermore, the MCG leaves may be considered to be a potent natural antioxidant material due to their high rates of radical scavenging effects compared to the stems and roots. The current data are similar to those of reported earlier for other crops [24,25]. Although the FRAP data did not observe significant differences in growth times and organs when compared to the radical inhibition results, their properties may be influenced by metabolite content through organs and environmental conditions during MCG growth [10,16,19,35]. Therefore, the MCG leaves collected on 31 May may be recommended as a potential material for developing functional foods and pharmaceutical products for humans because of their strong radical-scavenging inhibition and FRAP capacities. To our knowledge, for the first time, this study provides excellent information with regard to the variations and comparisons of the antioxidant properties in MCG organs with their maturation times.

In particular, we present the finding that the 50% ethanol extracts of MCG leaves exhibited potent capacities against DNA protection by hydroxyl radicals based on the earlier data using natural sources [26,32,39]. We are also confident that the DNA protection data for MCG leaves are associated with phenolic phytochemicals and other metabolites [14,25,26]. The DNA protection activities of MCG stems were realized at a dose-dependent concentration (50 → 1000 μg/mL) under gel mobility (Figure 2C,D), and their DNA protection rates exhibited low patterns compared to those of the MCG leaves. These findings may be significantly influenced by the difference in phenolic contents between leaves and stems, as suggested by the patterns of antioxidant properties against radicals [23,25]. As shown above, the MCG leaves possess the most predominant defense mechanisms for DNA damage, followed by stems and roots. Our investigations have revealed that the degrees of DNA protection in three organs at different maturation times were consistent with the order of antioxidant properties such as radical scavenging capacities and FRAP values. We are confident that the DNA protection ratios in the organs and maturation times of MCG may be remarkably affected by the phenolic content and profiles, as well as other metabolites [23,39]. MCG leaves can be considered to be a vital natural source for DNA protection in human health agency.

The increased rates of the fatty acid content in MCG may not be affected by environmental factors with the increase in maturation times. However, the metabolite concentrations are remarkably altered by the growth times and organs of natural plants, as reported previously [9,17,35,40]. Although the palmitic acid and linoleic acid distributions were consistent with those reported in the earlier literature on ginseng sprouts [15], their components exhibited significant variations in maturation times, which are considered factors related to food processing skills such as storage, aging, and fermentation, as well as other environmental factors [6,32,40,41]. Our data are consistent with previous studies that show that the content of linoleic acid was highest in the Panax species (*P. ginseng*, *P. notoginseng*, and *P. quinquefolius*) [15,42]. Generally, linoleic acid and linolenic acid play essential roles in plant defense mechanisms [18,42,43]. Moreover, the accumulation of linoleic acid is critically important in preventing infections caused by *Colletotrichum gloeosporioides* and *Botrytis cinerea* [18,43], as well as for reducing diverse pathogens and plant diseases in crops such as avocado and soybean [40,44]. Based on the above considerations, our findings will help promote the potential applications of MCG leaves to enhance properties that promote human health, providing important information regarding the optimal harvest times to maximize fatty acid content. Thus, the MCG samples harvested in May could be excellent materials for the development of human functional foods, owing to their high fatty acid content [9,40,42].

The present results regarding amino acids may be similar to the previous data, in that their contents are influenced by growth times, organs, genetics, and other environmental conditions [11,15,17]. Although arginine was observed to be the most abundant component of ginseng sprouts, followed by GABA and aspartic acid [3], the current results exhibited considerable differences in the individual components with high contents (>80 mg/100 g; leaves: alanine and GABA; roots: arginine) in MCG organs. This phenomenon confirms that the amino acid profiles and concentrations in the organs of MCG may be markedly affected by growth times [17,31]. Previous research indicated that arginine production is correlated with the ornithine cycle under environmental conditions [17]. However, our data might not be considerably associated with their relationship during the maturation times of MCG. Our data implies that amino acid profiles and concentrations in MCG leaves may depend on maturity times, as indicated in the previous literature [23,27]. Thus, MCG leaves may be considered to be a potential candidate source for nutraceutical agents, owing to their high amino acid content [11,17,30].

The individual ginsenoside showed strong variations in MCG leaves during different maturation times. Their differences implied that the ginsenoside accumulation rates in maturation periods may be influenced by the environmental states, as well as the conversion and biosynthesis of phytochemicals in the organs, as reported previously [2,3,5,10]. Furthermore, the above phenomena suggest that the ginsenoside profiles and accumulations may be markedly influenced by the biochemical modifications through the metabolite pathway mechanism in MCG during growth times [12,19,22,30]. It was also assumed that the ginsenoside derivatives were determined by the intense cellular division of growth periods, as well as by biosynthesis in organs and states of MCG, as previously reported for other crops [23,36]. Our results were similar to those from previous research, showing high ginsenoside content, including Re and Rg1 in the stem leaves of panax ginseng [5], and high elevated levels of ginsenoside Re in the leaves and roots of hydroponic-cultured ginseng [3]. The current data are similar to previous results that state that the growth times and organs of ginseng plants have considerable properties in ginsenoside content [2,3,5,10]. We believe that the MCG leaves harvested on 31 May are an excellent potential candidate source, and represent the optimal harvest time to maximize the ginsenoside content for the development of nutraceutical and functional food agents. To our knowledge, we presented the first quantitative data in relation to the ginsenoside content of MCG organs at different growth times.

Even though the individual and total phenolic contents were detected to have partial differences when compared to the previous literature [6], the order of phenolic components showed similar patterns. Considering the research findings, the phenolic phytochemical content may be positively correlated with growth conditions, genetics, and organs, as well as molecular figurations through their aromatic hydroxylation [27,30,36]. Our results indicate that the phenolic accumulations may be associated with the intense cellular division observed in natural plants during growth times [23,38]. The phenolic types and content may also be significantly influenced by their conversion and biosynthesis in the growth times of natural plants [27,31,34]. Overall, the appropriate harvest time for MCG to yield the highest phenolic metabolites appears to be after the sowing, as indicated by the 31 May sample. Also, our observations were coincident with those of the ginsenoside content. The present study suggests that MCG leaves may be utilized as a valuable resource in the food industry for human health due to their high phenolic phytochemicals. Therefore, this research may contribute to increasing the value of MCG leaves in the development of new functional foods using ginseng. Our study is the first to assess, compare, and quantify the phenolic phytochemicals in MCG organs.

## 5. Conclusions

The present study is the first to demonstrate the changes in metabolite constituents and antioxidant properties from different organs of the MCG plant during maturation times. Four metabolites exhibited remarkable differences between organs and maturation times. The MCG leaves displayed the predominant average metabolites, especially the harvested leaves on 31 May, which exhibited the most abundant content, with 1057.9 mg/100 g (fatty acid), 1153.3 mg/100 g (amino acid), 88.7 mg/g (ginsenoside), and 638.3 μg/g (phenolic phytochemical), respectively. Also, high ratios of palmitic acid, linoleic acid, and linolenic acid (fatty acids); alanine and γ-aminobutyric acid (amino acids); ginsenoside Re, ginsenoside Rd, and ginsenoside F2 (ginsenosides); and quercetin (phenolic phytochemicals) were observed during maturation times. The antioxidant capacities varied significantly in the MCG organs, compared to those in the maturation times, in the following order: leaves > stems > roots, with average effects of 77.9 > 39.1 > 8.4% (DPPH), 83.0 > 59.3 > 31.9% (ABTS), and 1.68 > 0.46 > 0.27 OD593 nm (FRAP) at 500 μg/mL. The DNA protection ratios were observed to show similar patterns as other antioxidant results, with the collected leaves on 31 May showing the highest protection at a concentration of 50 μg/mL, achieving 100%. In addition, the variability in metabolites were observed as follows: ginsenosides (83.3%) > amino acids (71.8%) > phenolic phytochemicals (61.1%) > fatty acids (58.8%), with each organ displaying significant differences and high relevance in metabolites and their compositions at maturation times. Our findings suggest that MCG leaves harvested on 31 May could be recommended as a potential material for developing functional food agents due to their high metabolite content and strong antioxidant capacities. In the future, more studies are needed to demonstrate in more detail the human-beneficial benefits of MCG leaves for potential pharmaceutical applications.

## Figures and Tables

**Table 1:** Variation of fatty acid content in different organs of MCG plants at maturation times.

Content (mg/100 g) ^a^	Maturation Times/Organs												
	17 May			31 May			21 June			13 July			
	Leaves	Stems	Roots	Leaves	Stems	Roots	Leaves	Stems	Roots	Leaves	Stems	Roots	
Saturated fatty acids													
Myristic acid (C14:0)	11.3 ± 0.1 b	11.0 ± 0.3 b	12.7 ± 0.1 a	7.6 ± 0.2 c	7.8 ± 0.1 c	11.3 ± 0.1 b	7.5 ± 0.1 c	4.2 ± 0.0 f	6.0 ± 0.1 d	11.2 ± 0.2 b	4.0 ± 0.0 f	5.6 ± 0.1 e	
Palmitic acid (C16:0)	240.6 ± 3.2 a	183.9 ± 1.8 e	221.0 ± 2.2 b	242.9 ± 1.9 a	186.2 ± 1.8 e	197.6 ± 2.1 c	225.1 ± 2.0 b	149.0 ± 1.5 f	119.0 ± 1.3 h	190.1 ± 1.5 d	127.9 ± 1.1 g	100.2 ± 1.5 i	
Stearic acid (C18:0)	52.2 ± 0.5 h	58.6 ± 0.6 f	62.4 ± 0.6 e	63.0 ± 1.5 d	45.1 ± 0.9 b	66.1 ± 1.2 c	69.5 ± 0.7 b	53.6 ± 0.5 g	54.8 ± 0.6 g	75.5 ± 0.7 a	54.3 ± 0.3 g	39.3 ± 0.5 i	
Arachidic acid (C20:0)	1.7 ± 0.0 i	nd	10.4 ± 0.1 a	2.3 ± 0.0 g	nd	8.3 ± 0.1 b	3.7 ± 0.0 e	nd	3.2 ± 0.0 f	5.9 ± 0.1 c	2.0 ± 0.0 h	3.8 ± 0.0 d	
Behenic acid (C22:0)	4.8 ± 0.1 g	4.8 ± 0.1 g	16.3 ± 0.2 a	4.6 ± 0.1 h	5.4 ± 0.1 f	12.4 ± 0.2 b	5.4 ± 0.0 f	3.4 ± 0.0 j	5.9 ± 0.1 e	6.7 ± 0.1 d	4.1 ± 0.0 i	7.0 ± 0.0 c	
Lignoceric acid (C24:0)	5.6 ± 0.1 e	6.8 ± 0.1 d	10.3 ± 0.1 a	5.0 ± 0.1 g	7.6 ± 0.2 c	8.3 ± 0.1 b	5.6 ± 0.0 e	2.6 ± 0.0 i	3.1 ± 0.0 i	5.3 ± 0.0 f	2.9 ± 0.0 i	3.5 ± 0.0 h	
Sum	316.2	265.1	333.1	325.4	252.1	304.0	316.8	212.8	192.0	294.7	195.2	159.4	
Unsaturated fatty acids													
Palmitoleic acid (C16:1)	nd ^b^	nd	nd	nd	nd	nd	nd	2.3 ± 0.0 a	nd	2.4 ± 0.0 a	nd	nd	
Oleic acid (C18:1n9c)	28.8 ± 0.3 k	34.5 ± 0.4 i	82.6 ± 0.8 f	100.0 ± 2.0 e	41.9 ± 0.8 h	72.7 ± 0.7 g	105.7 ± 1.0 d	186.9 ± 2.0 b	31.8 ± 0.3 j	188.5 ± 1.3 a	138.8 ± 1.5 c	42.8 ± 0.0 h	
Linoleic acid (C18:2n6c)	241.0 ± 2.4 h	277.1 ± 2.9 f	476.1 ± 4.8 a	274.1 ± 4.5 f	361.3 ± 3.4 d	440.7 ± 2.5 b	224.1 ± 2.2 i	410.6 ± 4.3 c	192.3 ± 2.5 j	251.9 ± 3.2 g	322.1 ± 3.3 e	188.4 ± 2.3 k	
γ-Linolenic acid (C18:3n6)	nd	nd	11.7 ± 0.1 a	nd	nd	7.80 ± 0.1 b	nd	nd	nd	nd	nd	nd	
α-Linolenic acid (C18:3n3)	294.0 ± 2.9 b	58.6 ± 0.6 e	29.2 ± 0.3 g	345.2 ± 6.9 a	80.3 ± 0.8 d	33.7 ± 0.5 f	126.3 ± 1.5 c	30.6 ± 0.3 g	14.4 ± 0.2 i	81.8 ± 0.8 d	33.5 ± 0.5 f	17.4 ± 0.1 h	
Eicosenoic acid (C20:1)	nd	nd	nd	nd	4.6 ± 0.0 a	4.1 ± 0.1 b	nd	3.9 ± 0.0 c	nd	nd	2.9 ± 0.0 d	nd	
Eicosadienoic acid (C20:2)	nd	nd	6.6 ± 0.1 a	nd	nd	6.1 ± 0.1 b	nd	nd	2.7 ± 0.0 c	nd	nd	2.8 ± 0.0 c	
Eicosatrienoic acid (C20:3n-3)	5.7 ± 0.1 c	nd	8.5 ± 1.2 a	5.1 ± 0.1 f	nd	6.6 ± 0.1 b	5.9 ± 0.1 c	nd	nd	5.5 ± 0.0 d	nd	nd	
Arachidonic acid (C20:4n6)	nd	nd	nd	1.7 ± 0.1 b	nd	nd	2.2 ± 0.0 a	nd	1.8 ± 0.0 b	2.3 ± 0.0 a	nd	1.8 ± 0.0 b	
Tricosanoic acid (20:4n6)	3.1 ± 0.1 b	nd	nd	3.3 ± 0.0 b	nd	5.7 ± 0.0 a	2.8 ± 0.0 c	nd	nd	nd	nd	2.4 ± 0.0 d	
Sum	575.5	370.2	621.8	732.5	488.1	584.3	470.5	634.3	246.3	536.5	497.3	255.6	
Total fatty acids	891.7	635.3	954.9	1057.9	740.2	888.3	787.3	847.1	438.3	831.2	692.5	415.0	

^a^ All values are presented as the mean ± SD, and differences were analyzed using Duncan’s multiple range test, *n* = 5. The results without common superscript letters (a–k) were statistically different (*p* < 0.05). ^b^ nd: not detected.

**Table 2:** Variation of amino acid content in different organs of MCG plants at maturation times.

Content (mg/100 g) ^a^	Maturation Times/Organs											
	17 May			31 May			21 June			13 July		
	Leaves	Stems	Roots	Leaves	Stems	Roots	Leaves	Stems	Roots	Leaves	Stems	Roots
Non-essential amino acids												
Phosphoethanolamine	nd ^b^	nd	47.3 ± 1.5 c	74.9 ± 3.5 a	nd	nd	62.0 ± 2.8 b	nd	nd	64.6 ± 3.6 b	nd	nd
Proline	31.4 ± 1.5 b	15.6 ± 0.9 d	16.5 ± 0.7 d	39.9 ± 0.9 a	12.3 ± 0.4 g	11.4 ± 0.3 h	31.2 ± 1.2 b	10.3 ± 0.2 i	9.0 ± 0.1 j	24.9 ± 1.2 c	13.2 ± 0.5 f	14.3 ± 0.3 e
Aspartic acid	23.6 ± 0.7 h	79.1 ± 3.8 b	30.9 ± 1.3 f	54.9 ± 2.6 c	48.8 ± 2.5 d	12.5 ± 0.4 j	49.7 ± 2.1 d	36.8 ± 1.4 e	10.2 ± 0.2 k	177.0 ± 16.3 a	28.9 ± 0.6 g	17.1 ± 0.4 i
Serine	21.5 ± 0.3 h	41.0 ± 2.5 d	24.2 ± 0.9 f	60.1 ± 2.4 b	28.3 ± 1.1 e	14.9 ± 0.3 k	47.8 ± 2.3 c	19.4 ± 0.7 i	11.7 ± 0.2 l	95.2 ± 4.0 a	22.8 ± 0.5 g	17.7 ± 0.7 j
Aspartic acid-NH_2_	28.5 ± 0.5 d	64.0 ± 2.6 b	35.1 ± 1.4 c	32.5 ± 0.9 d	32.8 ± 1.3 cd	15.8 ± 0.7 f	34.3 ± 1.7 c	18.7 ± 0.6 e	9.6 ± 0.3 i	324.9 ± 31.2 a	10.5 ± 0.2 h	14.0 ± 0.5 g
Glutamic acid	21.8 ± 0.4 e	35.3 ± 1.5 c	29.4 ± 1.3 d	39.2 ± 1.2 b	17.7 ± 0.5 f	13.1 ± 0.2 g	40.6 ± 2.0 b	13.4 ± 0.7 g	8.5 ± 0.3 j	118.9 ± 15.7 a	11.3 ± 0.2 h	10.2 ± 0.4 i
Sarcosine	1.3 ± 0.1 b	nd	nd	0.7 ± 0.0 c	nd	nd	6.1 ± 0.2 a	nd	nd	nd	nd	nd
Aminoadipic acid	4.4 ± 0.1 d	0.7 ± 0.0 h	1.4 ± 0.2 f	2.0 ± 0.3 e	1.4 ± 0.1 f	nd	6.3 ± 0.2 b	1.1 ± 0.1 g	nd	20.4 ± 0.7 a	5.1 ± 0.2 c	0.7 ± 0.0 h
Glycine	15.7 ± 0.2 b	6.3 ± 0.7 h	10.0 ± 0.6 e	12.2 ± 0.5 d	5.9 ± 0.3 i	7.5 ± 0.2 g	14.2 ± 0.4 c	9.5 ± 0.2 f	5.7 ± 0.2 i	17.7 ± 0.3 a	10.5 ± 0.1 e	6.7 ± 0.3 h
Alanine	84.1 ± 3.9 c	28.8 ± 1.2 e	45.2 ± 2.4 d	125.3 ± 10.6 a	23.8 ± 0.7 f	28.7 ± 0.6 e	128.6 ± 11.1 a	23.1 ± 0.8 g	23.2 ± 1.1 g	100.0 ± 9.8 b	21.2 ± 0.5 h	24.9 ± 0.9 f
Citrulline	nd	nd	3.0 ± 0.3 a	nd	nd	1.6 ± 0.1 b	nd	nd	nd	2.7 ± 0.1 a	nd	nd
α-aminobutyric acid	41.5 ± 1.5 b	56.3 ± 2.2 a	14.6 ± 0.6 f	10.9 ± 0.3 g	30.6 ± 1.1 c	4.1 ± 0.3 i	24.0 ± 1.0 e	24.8 ± 0.6 e	2.5 ± 0.1 j	10.9 ± 0.2 g	20.4 ± 0.3 d	6.7 ± 0.3 h
Cystine	nd	nd	nd	4.3 ± 0.2 b	nd	3.3 ± 0.3 c	nd	nd	2.2 ± 0.1 d	5.1 ± 0.1 a	nd	2.2 ± 0.2 d
Cystathionine	nd	41.4 ± 2.4 a	nd	nd	25.4 ± 0.8 b	nd	nd	14.0 ± 0.4 c	nd	nd	10.4 ± 0.2 d	nd
Tyrosine	51.3 ± 1.9 a	18.1 ± 1.1 d	22.5 ± 0.8 c	39.3 ± 10.1 b	13.2 ± 0.6 e	12.7 ± 0.7 f	21.1 ± 0.8 c	10.6 ± 0.4 g	9.1 ± 0.3 h	12.7 ± 0.3 f	18.7 ± 0.5 d	13.4 ± 0.7 e
β-alanine	22.5 ± 0.7 b	24.6 ± 1.3 a	19.2 ± 0.7 c	8.3 ± 0.4	13.4 ± 0.4 e	5.8 ± 0.2 i	11.5 ± 0.5 f	9.7 ± 0.3 g	5.2 ± 0.2 i	13.7 ± 0.2 e	17.2 ± 0.5 d	8.5 ± 0.6 h
β-aminoisobutyric acid	26.0 ± 0.5 a	12.1 ± 0.5 e	9.8 ± 0.3 f	15.1 ± 0.5 d	4.1 ± 0.3 g	2.9 ± 0.1 i	21.7 ± 0.7 b	3.6 ± 0.2 h	3.5 ± 0.2 h	18.9 ± 0.6 c	3.9 ± 0.1 g	4.0 ± 0.3 g
γ-aminobutyric acid	106.7 ± 6.5 d	44.5 ± 1.6 h	94.5 ± 5.9 e	158.0 ± 10.8 c	34.9 ± 1.2 j	60.4 ± 1.5 f	181.5 ± 13.2 b	37.4 ± 2.0 i	61.8 ± 2.7 f	211.7 ± 19.5 a	23.0 ± 0.8 k	52.5 ± 2.3 g
Aminoethanol	17.6 ± 0.3 b	6.8 ± 0.3 fg	11.0 ± 0.6 e	14.6 ± 0.3 d	6.6 ± 0.3 g	7.3 ± 0.9 f	15.4 ± 0.5 c	5.6 ± 0.2 h	6.9 ± 0.6 fg	19.0 ± 0.5 a	4.8 ± 0.1 i	6.4 ± 0.2 g
Hydroxylysine	1.8 ± 0.1 a	1.3 ± 0.4 bc	2.0 ± 0.2 a	1.8 ± 0.1 a	1.4 ± 0.1 bc	1.6 ± 0.3 b	1.5 ± 0.1 b	1.3 ± 0.1 c	1.5 ± 0.2 b	nd	1.2 ± 0.1 c	1.5 ± 0.1 b
Ornithine	16.2 ± 0.2 a	4.5 ± 0.7 g	7.5 ± 0.3 c	11.2 ± 0.4 b	6.6 ± 0.2 d	5.5 ± 0.5 e	4.4 ± 0.1 g	7.4 ± 0.2 c	4.8 ± 0.3 f	5.0 ± 0.2 f	6.5 ± 0.3 d	5.3 ± 0.3 e
Arginine	79.4 ± 3.8 d	21.2 ± 0.6 j	382.4 ± 25.3 a	37.5 ± 0.9 f	27.6 ± 0.7 h	194.0 ± 16.0 b	33.9 ± 0.8 g	23.0 ± 1.1 i	140.0 ± 11.1	50.6 ± 1.3 e	25.1 ± 0.9 h	89.4 ± 4.1 c
Total	595.3	501.6	806.5	742.7	334.8	403.1	735.8	269.7	315.4	1293.9	254.7	295.5
Essential amino acids												
Threonine	55.8 ± 2.5 b	25.3 ± 0.7 e	25.0 ± 0.5 e	41.3 ± 1.3 d	20.2 ± 0.4 f	15.1 ± 0.5 g	41.8 ± 1.2 c	15.6 ± 0.6 g	12.2 ± 0.4 i	69.9 ± 2.7 a	14.2 ± 0.4 h	15.3 ± 1.1 g
Valine	37.5 ± 1.4 f	47.5 ± 2.3 d	39.1 ± 0.9 e	72.2 ± 3.8 c	40.4 ± 0.9 e	22.1 ± 0.7 h	78.2 ± 3.3 b	34.8 ± 1.2 g	16.3 ± 0.2 i	136.0 ± 10.1 a	12.3 ± 0.2 j	10.4 ± 0.7 k
Methionine	37.6 ± 1.6 a	nd	12.8 ± 0.1 e	22.8 ± 0.7 c	nd	6.6 ± 0.3 f	27.8 ± 1.3 b	5.4 ± 0.2 g	4.6 ± 0.1 h	18.3 ± 0.3 d	nd	5.4 ± 0.2 g
Isoleucine	75.2 ± 2.8 b	26.9 ± 1.3 e	25.8 ± 0.0 e	50.2 ± 2.6 d	19.9 ± 0.5 g	13.9 ± 0.3 i	65.3 ± 2.5 c	17.0 ± 0.5 h	9.7 ± 0.2 j	105.3 ± 9.7 a	24.2 ± 0.7 f	13.3 ± 0.5 i
Leucine	52.0 ± 2.3 c	25.5 ± 1.4 e	36.0 ± 1.4 d	77.1 ± 3.2 b	23.7 ± 0.7 f	23.3 ± 0.5 f	80.3 ± 2.7 b	21.0 ± 0.4 e	16.5 ± 0.6 g	132.8 ± 10.5 a	12.7 ± 0.4 h	15.4 ± 0.2 g
Phenylalanine	46.6 ± 1.5 d	21.0 ± 0.9 f	29.4 ± 0.7 e	64.9 ± 2.4 c	13.8 ± 0.4 g	15.7 ± 0.7 f	73.0 ± 2.0 b	12.7 ± 0.5 h	12.0 ± 0.7 h	127.6 ± 12.4 a	11.5 ± 0.6 h	10.3 ± 0.2 i
Lysine	50.1 ± 1.4 c	13.8 ± 0.7 f	37.8 ± 1.2 d	67.7 ± 2.3 b	18.4 ± 0.3 e	23.7 ± 0.7	68.9 ± 1.4 b	16.9 ± 0.3 f	14.1 ± 0.5 g	77.3 ± 3.8 a	18.6 ± 1.0 e	11.8 ± 0.3 h
Histidine	15.1 ± 0.7 b	4.9 ± 0.3 g	15.5 ± 0.6 b	14.4 ± 0.5 c	4.9 ± 0.1 g	8.9 ± 0.3 e	11.4 ± 0.4 d	4.6 ± 0.1 g	5.7 ± 0.2 f	28.1 ± 1.2 a	11.3 ± 0.8 d	5.4 ± 0.2 f
Total	369.9	164.9	221.4	410.6	141.3	129.3	446.7	128.0	91.1	695.3	104.8	87.3
Total amino acids	965.2	666.5	1027.9	1153.3	476.1	532.4	1182.5	397.7	406.5	1989.2	359.5	382.8

^a^ All values are presented as the mean ± SD, and differences were analyzed using Duncan’s multiple range test, *n* = 5. The results without common superscript letters (a–k) were statistically different (*p* < 0.05). ^b^ nd: not detected.

**Table 3:** Variation of ginsenoside contents in different organs of MCG plants at maturation times.

Content (mg/g) ^a^	Maturation Times/Organs											
	17 May			31 May			21 June			13 July		
	Leaves	Stems	Roots	Leaves	Stems	Roots	Leaves	Stems	Roots	Leaves	Stems	Roots
Protopanaxtriol												
Ginsenoside Rg1 (1)	4.5 ± 0.8 c	1.0 ± 0.1 h	3.2 ± 0.2 d	6.2 ± 0.5 a	1.4 ± 0.5 g	2.6 ± 0.6 e	5.6 ± 0.4 b	1.2 ± 0.2 g	1.3 ± 0.2 g	5.5 ± 0.3 b	1.3 ± 0.4 g	1.9 ± 0.3 f
Ginsenoside Re (2)	19.1 ± 1.2 a	2.3 ± 0.3 f	5.7 ± 0.4 c	18.2 ± 1.4 a	4.9 ± 0.9 d	4.2 ± 0.8 d	17.3 ± 1.3 b	4.8 ± 0.3 d	2.6 ± 0.2 f	16.9 ± 1.2 b	3.6 ± 0.7 e	3.3 ± 0.4 e
Ginsenoside Rf (4)	0.2 ± 0.0 d	0.2 ± 0.0 d	1.2 ± 0.1 a	0.2 ± 0.0 d	0.2 ± 0.0 d	0.9 ± 0.1 b	0.2 ± 0.0 d	0.2 ± 0.0 d	0.4 ± 0.0 c	0.1 ± 0.0 d	0.2 ± 0.0 d	0.6 ± 0.0 c
Ginsenoside F5 (5)	2.0 ± 0.2 a	nd ^b^	0.2 ± 0.0 d	2.4 ± 0.6 a	0.2 ± 0.0 d	0.5 ± 0.0 c	2.3 ± 0.6 a	0.1 ± 0.0 d	0.1 ± 0.0 d	1.9 ± 0.5 b	0.1 ± 0.0 d	0.2 ± 0.0 d
Ginsenoside F3 (6)	7.9 ± 0.5 a	0.4 ± 0.0 d	nd	7.2 ± 0.9 ab	0.9 ± 0.0 c	nd	7.6 ± 0.8 a	0.7 ± 0.0 d	nd	6.9 ± 0.9 b	0.6 ± 0.1 d	nd
Ginsenoside Rg2 (7)	1.5 ± 0.3 a	0.3 ± 0.0 d	0.7 ± 0.1 b	1.4 ± 0.1 a	0.4 ± 0.0 c	0.6 ± 0.0 b	1.5 ± 0.2 a	0.3 ± 0.0 d	0.2 ± 0.0 d	1.6 ± 0.1 a	0.2 ± 0.0 d	0.4 ± 0.0 c
Ginsenoside Rh1(8)	nd	nd	0.5 ± 0.0 a	nd	nd	0.6 ± 0.0 a	nd	nd	0.3 ± 0.0 b	nd	nd	0.5 ± 0.0 a
Ginsenoside F1 (11)	2.2 ± 0.3 b	0.2 ± 0.0 c	nd	3.0 ± 0.1 a	0.4 ± 0.0	nd	2.8 ± 0.3 a	0.3 ± 0.0 c	nd	3.1 ± 0.2 a	0.3 ± 0.0 c	nd
Protopanaxtriol (18)	0.4 ± 0.0 d	0.1 ± 0.0 e	0.1 ± 0.0 e	2.2 ± 0.2 b	0.6 ± 0.0 c	0.3 ± 0.0 d	0.5 ± 0.0 c	0.5 ± 0.0 c	0.2 ± 0.0 e	2.8 ± 0.2 a	0.6 ± 0.0 c	0.2 ± 0.0 e
Sum	37.8	4.5	11.6	40.8	9.0	9.7	37.8	8.1	5.1	38.8	6.9	7.1
Protopanaxdiol												
Ginsenoside Rb1 (9)	0.8 ± 0.0 e	nd	10.3 ± 0.7 a	0.9 ± 0.1 e	0.7 ± 0.0 f	9.5 ± 0.4 b	0.8 ± 0.1 e	0.6 ± 0.0 f	4.3 ± 0.7 d	0.8 ± 0.0 e	0.5 ± 0.0 g	7.6 ± 0.7 c
Ginsenoside Rc (10)	1.5 ± 0.3 d	nd	4.0 ± 0.3 a	2.9 ± 0.4 b	0.6 ± 0.0 e	3.7 ± 0.2 a	2.0 ± 0.2 c	0.5 ± 0.0 e	1.6 ± 0.4 d	1.9 ± 0.3 c	0.2 ± 0.0 f	2.9 ± 0.3 b
Ginsenoside Rb2 (12)	1.9 ± 0.2 e	nd	3.7 ± 0.3 b	4.3 ± 0.6 a	1.0 ± 0.2	2.8 ± 0.2 c	4.0 ± 0.7 a	0.7 ± 0.1 g	1.2 ± 0.1 f	2.7 ± 0.3 c	0.4 ± 0.0 h	2.3 ± 0.2 d
Ginsenoside Rb3 (13)	nd	nd	0.6 ± 0.0 b	0.8 ± 0.1 a	nd	0.5 ± 0.0 c	0.6 ± 0.0 b	nd	nd	0.8 ± 0.0 a	nd	0.4 ± 0.0 c
Ginsenoside Rd (14)	8.5 ± 0.7 b	1.3 ± 0.8 f	2.4 ± 0.1 d	14.8 ± 0.8 a	4.0 ± 0.6 c	2.6 ± 0.3 d	13.0 ± 1.2 a	2.8 ± 0.4 d	1.3 ± 0.1 f	8.7 ± 0.8 b	1.8 ± 0.5 e	2.0 ± 0.2 e
Ginsenoside Rd2 (15)	8.4 ± 0.9 a	0.2 ± 0.0 f	0.9 ± 0.1 e	7.6 ± 0.6 b	1.3 ± 0.2 d	1.4 ± 0.2 d	7.7 ± 0.8 b	1.4 ± 0.5 d	0.8 ± 0.1	7.0 ± 0.9 c	1.5 ± 0.3 d	1.4 ± 0.1 d
Ginsenoside F2 (16)	13.1 ± 0.9 a	0.5 ± 0.1 g	0.1 ± 0.0 h	10.1 ± 0.7 b	1.4 ± 0.1 d	0.5 ± 0.0 g	10.8 ± 0.6 b	1.1 ± 0.2 e	0.7 ± 0.0 f	9.5 ± 0.8 c	1.6 ± 0.3 d	0.7 ± 0.0 f
Ginsenoside Rg3 (17)	0.6 ± 0.0 b	0.1 ± 0.0 f	0.2 ± 0.0 d	0.5 ± 0.0 c	0.4 ± 0.0 c	0.3 ± 0.0 d	0.7 ± 0.0 b	0.7 ± 0.1 b	0.2 ± 0.0 d	1.5 ± 0.3 a	0.8 ± 0.1 b	0.2 ± 0.0 d
Compound K (19)	0.4 ± 0.0 d	0.1 ± 0.0 f	0.4 ± 0.0 d	0.9 ± 0.1 b	0.6 ± 0.0 c	0.4 ± 0.0 d	0.6 ± 0.0 c	0.5 ± 0.0 d	0.2 ± 0.0 f	1.2 ± 0.2 a	0.7 ± 0.1 c	0.3 ± 0.0 e
Ginsenoside Rh2 (20)	0.5 ± 0.0 e	0.1 ± 0.0 f	2.5 ± 0.2 a	0.4 ± 0.0 e	0.4 ± 0.0 e	1.9 ± 0.2 b	1.2 ± 0.1 d	0.4 ± 0.0 e	0.1 ± 0.0 f	1.1 ± 0.2 d	0.4 ± 0.0 e	1.4 ± 0.3 c
Protopanaxdiol (21)	1.5 ± 0.2 a	0.5 ± 0.1 c	0.4 ± 0.0 cf	1.0 ± 0.1 b	1.0 ± 0.1 b	0.4 ± 0.0 cf	0.9 ± 0.1 b	0.6 ± 0.0 c	0.3 ± 0.0 d	0.7 ± 0.1 bc	0.9 ± 0.1 b	0.4 ± 0.0 cf
Sum	37.2	2.8	25.5	44.2	11.4	24.0	42.3	9.3	10.7	35.9	8.8	19.6
Oleanane												
Ginsenoside Ro (3)	9.8 ± 0.7 a	0.9 ± 0.0 f	3.7 ± 0.6 b	3.7 ± 0.8 b	2.6 ± 0.5 c	2.2 ± 0.2 d	2.1 ± 0.3 d	0.5 ± 0.0 h	0.7 ± 0.1 g	3.6 ± 0.5 b	1.2 ± 0.2 e	0.4 ± 0.0 h
Sum	9.8	0.9	3.7	3.7	2.6	2.2	2.1	0.5	0.7	3.6	1.2	0.4
Total ginsenosides	84.8	8.2	40.8	88.7	23.0	35.9	82.2	17.9	16.5	78.3	16.9	27.1

^a^ All values are presented as the mean ± SD, and differences were analyzed using Duncan’s multiple range test, *n* = 5. The results without common superscript letters (a–h) were statistically different (*p* < 0.05). ^b^ nd: not detected.

**Table 4:** Variation of phenolic phytochemical content in different organs of MCG plants at maturation times.

Content (μg/g) ^a^	Maturation Times/Organs											
	17 May			31 May			21 June			13 July		
	Leaves	Stems	Roots	Leaves	Stems	Roots	Leaves	Stems	Roots	Leaves	Stems	Roots
Phenolic acids												
Gallic acid	6.8 ± 0.2 b	7.4 ± 0.9 a	4.9 ± 0.7 d	6.2 ± 0.1 b	5.5 ± 0.1 c	5.5 ± 0.3 c	5.5 ± 0.6 c	5.3 ± 0.3 c	5.2 ± 0.8 c	5.6 ± 0.6 c	5.3 ± 0.8 c	5.3 ± 0.4 c
Protocatechuic acid	6.0 ± 0.3 b	2.5 ± 0.3 e	0.5 ± 0.0 g	4.2 ± 0.2 c	3.5 ± 0.1 d	nd ^b^	1.9 ± 0.2 f	6.7 ± 0.4 a	nd	4.2 ± 0.3 c	4.9 ± 0.9 c	nd
Chlorogenic acid	57.9 ± 1.5 b	24.6 ± 1.3 d	19.9 ± 1.1 e	82.3 ± 2.8 a	18.1 ± 1.2 ef	12.7 ± 1.5 g	58.8 ± 3.4 b	12.3 ± 0.8 g	12.5 ± 0.9 g	46.4 ± 7.5 c	21.0 ± 1.3 e	12.1 ± 1.3 g
*p*-Hydroxybenzoic acid	36.0 ± 0.9 a	12.3 ± 0.9 e	2.1 ± 0.3 g	27.8 ± 1.3 b	10.9 ± 0.9	2.4 ± 0.7 f	18.6 ± 1.2 c	13.3 ± 0.7 e	1.6 ± 0.3 h	15.8 ± 1.4 d	12.2 ± 1.2 e	2.0 ± 0.4 f
Vanillic acid	nd	nd	nd	nd	1.1 ± 0.2 c	nd	3.7 ± 0.7 a	nd	nd	2.1 ± 0.5 b	0.3 ± 0.0 d	nd
*p*-Coumaric acid	2.0 ± 0.3 b	1.9 ± 0.4 b	nd	nd	3.8 ± 0.6 a	nd	4.0 ± 0.5 a	3.8 ± 0.3 a	nd	0.6 ± 0.1 d	1.0 ± 0.2 c	nd
Ferulic acid	4.0 ± 0.7 b	1.3 ± 0.1 e	nd	2.9 ± 0.1 d	0.7 ± 0.0 f	nd	3.9 ± 0.3 b	0.9 ± 0.1 f	0.4 ± 0.0 g	12.2 ± 0.9 a	3.6 ± 0.6 c	3.5 ± 0.3 c
Veratric acid	nd	nd	nd	8.8 ± 0.2 b	4.0 ± 0.5 c	2.0 ± 0.4 d	10.8 ± 0.7 a	4.8 ± 0.5 c	1.8 ± 0.2 d	nd	nd	nd
Benzoic acid	nd	nd	nd	nd	nd	nd	nd	nd	nd	nd	nd	nd
*t*-Cinnamic acid	1.7 ± 0.1 c	1.8 ± 0.1 c	1.1 ± 0.2 d	0.5 ± 0.0 f	0.9 ± 0.1 e	0.6 ± 0.0 f	0.5 ± 0.0 f	1.3 ± 0.3 d	0.6 ± 0.0 f	2.3 ± 0.5 b	1.8 ± 0.2 c	2.9 ± 0.2 a
Total phenolic acids	114.4	51.8	28.4	132.7	48.5	23.2	107.7	48.4	22.2	89.2	50.1	25.8
Flavonols												
Epigallocatechin	52.2 ± 2.5 c	51.5 ± 2.5 c	36.3 ± 1.3 d	76.4 ± 1.9 b	90.6 ± 3.9 a	25.2 ± 1.2 e	77.8 ± 3.9 b	56.2 ± 2.3 c	23.9 ± 1.3 e	54.2 ± 3.9 c	31.7 ± 3.6 d	26.6 ± 2.3 e
Catechin	125.5 ± 9.3 a	27.1 ± 1.3 e	11.0 ± 0.6 g	99.4 ± 2.5 d	11.5 ± 0.7 g	5.4 ± 0.3 h	119.2 ± 8.7 b	13.4 ± 0.7 f	4.4 ± 0.9 h	102.9 ± 12.0 c	30.0 ± 3.5 e	1.4 ± 0.5 i
Epicatechin	48.9 ± 1.6 b	20.2 ± 1.4 c	10.6 ± 0.7 f	80.5 ± 3.1 a	3.1 ± 0.2 h	1.8 ± 0.2 i	13.4 ± 1.1 e	17.1 ± 0.9 d	1.8 ± 0.4 i	17.2 ± 1.2 d	5.8 ± 0.7 g	5.0 ± 0.9 g
Epigallocatechin gallate	nd	nd	4.9 ± 0.2 d	28.0 ± 0.9 a	11.7 ± 0.6 c	3.1 ± 0.2 e	14.9 ± 1.2 b	nd	3.0 ± 0.7 e	14.5 ± 0.8 b	15.2 ± 1.2 b	3.0 ± 0.3 e
Vanillin	nd	nd	nd	nd	nd	nd	nd	nd	nd	nd	nd	nd
Rutin	6.3 ± 0.2 c	2.2 ± 0.2 d	2.4 ± 0.3 d	nd	2.1 ± 0.3 d	1.3 ± 0.1 e	8.1 ± 0.9 b	6.2 ± 0.6 c	1.0 ± 0.2 e	17.7 ± 1.3 a	1.9 ± 0.6 d	0.8 ± 0.1 f
Catechin gallate	8.0 ± 0.3 b	4.1 ± 0.3 e	3.2 ± 0.5 e	5.5 ± 0.3 d	3.8 ± 0.5 e	2.7 ± 0.3 ef	7.5 ± 0.7 c	3.1 ± 0.5 ef	2.5 ± 0.7 f	9.3 ± 0.7 a	4.7 ± 0.9 cd	2.6 ± 0.7 f
Quercetin	131.7 ± 8.7 c	100.9 ± 9.1 e	82.4 ± 3.9 f	197.9 ± 12.0 a	113.4 ± 7.1 d	107.9 ± 8.5 d	177.7 ± 13.9 b	119.1 ± 11.3 d	99.1 ± 9.3 e	172.6 ± 14.3 b	128.7 ± 16.5 c	99.7 ± 12.7 e
Naringin	17.4 ± 0.9 a	7.4 ± 0.6 c	2.4 ± 0.3 e	3.7 ± 0.2 d	nd	1.0 ± 0.0 f	3.7 ± 0.5 d	0.8 ± 0.1 f	nd	12.9 ± 0.6 b	7.4 ± 0.8 c	2.1 ± 0.4 e
Naringenin	15.2 ± 0.7 d	34.5 ± 1.4 a	23.3 ± 1.0 b	7.8 ± 0.2 f	20.3 ± 1.3 c	4.3 ± 0.6 g	10.3 ± 0.7 e	25.6 ± 0.9 b	4.2 ± 0.8 g	14.0 ± 0.7 d	7.1 ± 0.9 f	7.9 ± 0.5 f
Formonoetin	5.2 ± 0.5 b	3.1 ± 0.3 d	3.8 ± 0.2 d	6.4 ± 0.1 a	4.8 ± 0.8 c	2.5 ± 0.3 e	7.2 ± 0.6 a	6.8 ± 0.7 a	2.7 ± 0.5 e	5.6 ± 0.8 b	2.9 ± 0.5 e	2.6 ± 0.7 e
Total flavonols	410.4	251.0	180.3	505.6	261.3	155.2	440.1	248.2	142.6	420.9	235.4	151.7
Total phenolics	524.8	302.8	208.7	638.3	309.8	178.4	547.8	296.6	164.8	510.1	285.5	177.5

^a^ All values are presented as the mean ± SD, and differences were analyzed using Duncan’s multiple range test, *n* = 5. The results without common superscript letters (a–i) were statistically different (*p* < 0.05). ^b^ nd: not detected.

## Data Availability

The original contributions presented in the study are included in the article/Supplementary Materials. Further inquiries can be directed to the corresponding author.

## Supplementary Materials

The following supporting information can be downloaded at: https://www.mdpi.com/article/10.3390/antiox13050612/s1, Figure S1 General appearances of MCG samples in different maturation times. Figure S2. Changes in weight ratio of MCG organs at maturation times.
