# Supplementary material for: Examining the Alterations in Metabolite Constituents and Antioxidant Properties in Mountain-Cultivated Ginseng (Panax ginseng C.A. Meyer) Organs during a Two-Month Maturation Period
Source: Antioxidants (Basel). 2024 May 17;13(5):612. doi: 10.3390/antiox13050612 (PMC11117551; doi:10.3390/antiox13050612)
Supplement: Supplementary file 1 [file antioxidants-13-00612-s001.zip › antioxidants-2982184-supplementary.pdf]

# Supplementary material

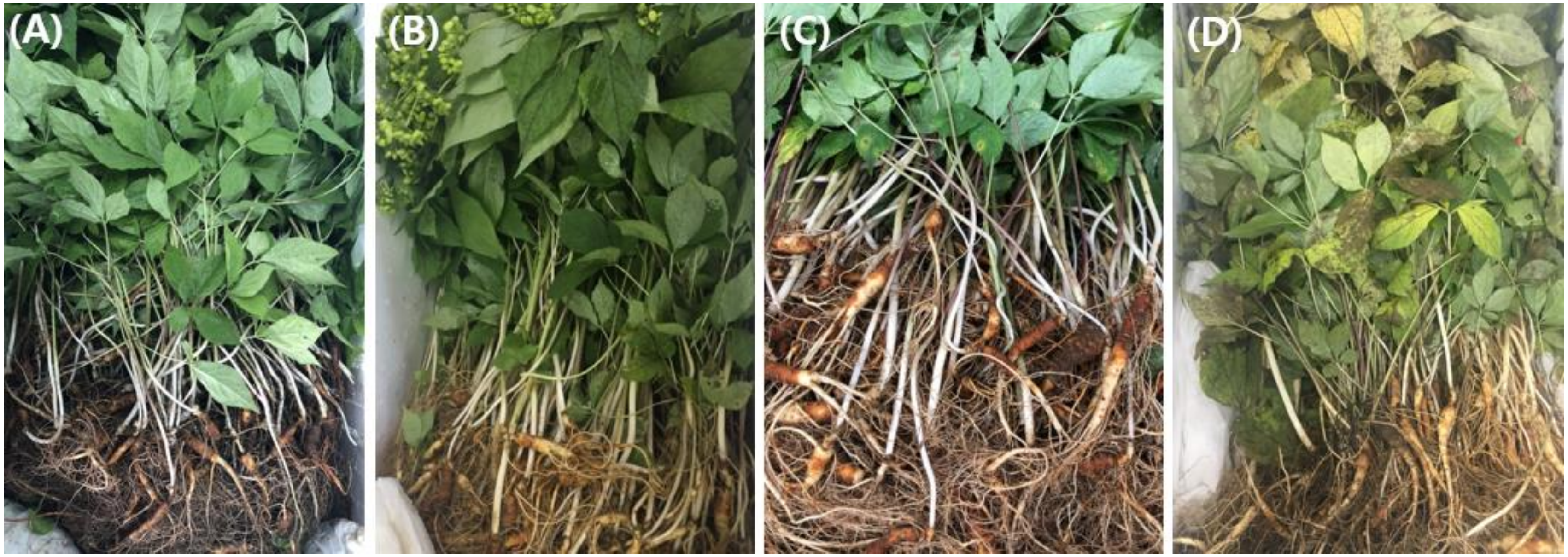


**Figure S1.** General appearances of MCG samples in different maturation times. (A) 17 May sample (2017) (B) 31 May sample (2017) (C) 21 June sample (2017) (D) 13 July sample (2017)

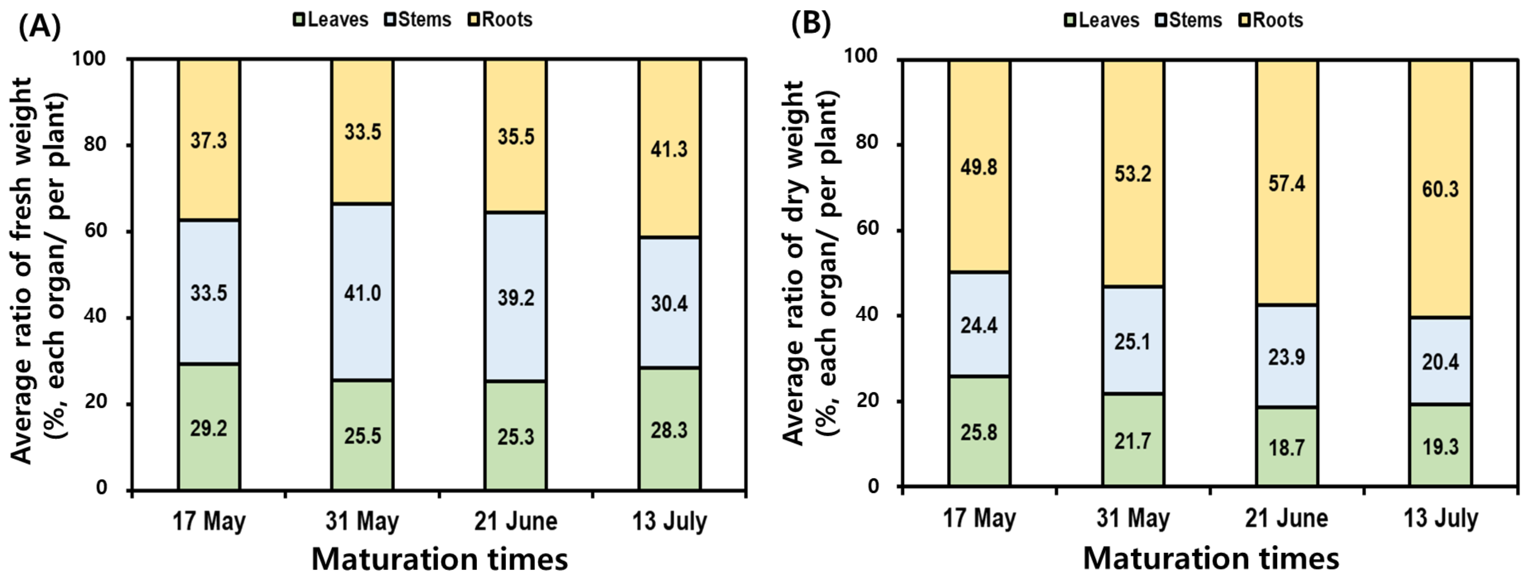


**Figure S2.** Changes in weight ratio of MCG organs at maturation times. (A) average ratio of fresh weight and (B) average ratio of dry weight. All experiments were repeated with a hundred MCG.
